# Global trend of diabetes mortality attributed to vascular complications, 2000–2016

**DOI:** 10.1186/s12933-020-01159-5

**Published:** 2020-10-20

**Authors:** Wei Ling, Yi Huang, Yan-Mei Huang, Rong-Rong Fan, Yi Sui, Hai-Lu Zhao

**Affiliations:** 1 Department of Endocrinology, Xiangya Hospital, Central South University, Changsha, 410008 China; 2 Center for Diabetic Systems Medicine, Guangxi Key Laboratory of Excellence, Guilin Medical University, Guilin, 541100 China; 3 Department of Immunology, Guangxi Area of Excellence, Guilin Medical University, Guilin, 541100 China; 4 Department of Geriatrics, Zhongshan Hospital, Fudan University, Shanghai, 200032 China; 5 Department of Biosciences and Nutrition, Karolinska Institute, 171 77 Stockholm, Sweden; 6 Department of Clinical Nutrition, The First Affiliated Hospital of Sun Yat-Sen University, Guangzhou, 510080 China

**Keywords:** Diabetes mellitus, Vascular complication, Diabetic nephropathy, Mortality

## Abstract

**Background:**

The global epidemic of diabetes mellitus continues to grow and affects developed and developing countries alike. Intensive glycemic control is thought to modify the risks for vascular complications, hence the risks for diabetes-related death. We investigated the trend of diabetic vascular complication-related deaths between 2000 and 2016 in the global diabetes landscape.

**Methods:**

We collected 17 years of death certificates data from 108 countries in the World Health Organization mortality database between 2000 and 2016, with coding for diabetic complications. Crude and age-standardized proportions and rates were calculated. Trend analysis was done with annual average percentage change (AAPC) of rates computed by joinpoint regression.

**Results:**

From 2000 through 2016, 7,108,145 deaths of diabetes were reported in the 108 countries. Among them, 26.8% (1,904,787 cases) were attributed to vascular complications in damaged organs, including the kidneys (1,355,085 cases, 71.1%), peripheral circulatory (515,293 cases, 27.1%), nerves (28,697 cases, 1.5%) and eyes (5751 cases, 0.3%). Overall, the age-standardized proportion of vascular complication-related mortality was 267.8 [95% confidence interval (95% CI), 267.5–268.1] cases per 1000 deaths and the rate was 53.6 (95% CI 53.5–53.7) cases per 100,000 person-years. Throughout the 17-year period, the overall age-standardized proportions of deaths attributable to vascular complications had increased 37.9%, while the overall age-standardized mortality rates related to vascular complications had increased 30.8% (AAPC = 1.9% [1.4–2.4%, p < 0.05]). These increases were predominantly driven by a 159.8% increase in the rate (AAPC = 2.7% [1.2–4.3%, p < 0.05]) from renal complications. Trends in the rates and AAPC of deaths varied by type of diabetes and of complications, as well as by countries, regions and domestic income.

**Conclusion:**

Diabetic vascular complication-related deaths had increased substantially during 2000–2016, mainly driven by the increased mortality of renal complications.

## Background

The global prevalence of diabetes mellitus (DM) has reached 463 million in 2019, and the World Health Organization (WHO) estimated that, worldwide, the number of people living with diabetes would increase to 700 million by 2045 [1]. Intensive glycemic control has been shown to be beneficial for the management of microvascular complications of diabetes [2, 3]. As a result, the prevalence of these diabetes-related complications might have also changed [4].

Diabetes has been implicated in all-cause mortality, especially in deaths related to cardiovascular and cerebral vascular disease. [5, 6] Additionally, diabetes is often associated with premature deaths from noncommunicable diseases [7] as well as communicable diseases, including SARS [8], MERS, H1N1 [9, 10] and the COVID-19 [11, 12]. In 2016, an estimated 1.6 million deaths were directly caused by diabetes. Another 2.2 million deaths were attributable to high blood glucose in 2012 [13]. Collectively, mortality of diabetes is often determined by prevalence of diabetes, clinical care, self-management behaviors and risk-factor control. Given that clinical guidelines and recommendations regarding some of these factors have been considerably modified during the past two decades, diabetes-related deaths from microvascular complications might have changed due to tight glycemic control. However, the global burden of vascular complication-related mortality remains unknown. Understanding the mortality trends of vascular complication-related events will help evaluate the current clinical hypoglycemic treatments and guide future diabetes management. Therefore, we aimed to investigate the global burden and trends of diabetic deaths due to vascular complications.

## Methods

### Data collection

We identified countries with available data of all-cause diabetes-related deaths from the WHO Mortality DataBase (WDB), which provides the most comprehensive (standardized national) mortality statistics for countries around the world [14]. The data from the WDB are official national statistics in the sense that they have been transmitted to WHO by the competent authorities of the countries concerned. The WDB comprises deaths registered in national vital registration systems, with underlying cause of death as coded by the relevant national authority. Underlying cause of death is defined as “the disease or injury which initiated the train of morbid events leading directly to death, or the circumstances of the accident or violence which produced the fatal injury” in accordance with the rules of the International Classification of Diseases (ICD). Therefore, there is primary cause-specific attribution of the ICD code to mortality in the death certificates. The WDB contains number of deaths by country, year, sex, age group and cause of death as far back from 1950. Data are included only for countries reporting data properly coded according to the ICD. We used ICD-10 (version 2019) with detailed codes (4th character, ICD-104) to identify all diabetes-related deaths (E10–E14 codes), which include deaths from type 1 DM (E10), type 2 DM (E11), malnutrition-related DM (E12), other specified DM (E13) and unspecified DM (E14). The ICD-10 was endorsed in May 1990 by the Forty-Third World Health Assembly. From 1990 to 2017, a total 10 editions of ICD-10 were released by WHO with the latest edition released in 2016 [15]. We compared all ICD-10 editions and found consistent codes for diabetes mellitus and diabetic complications (E10–E14) during this period. For countries with available ICD codes, we stratified the data by diabetic complications. Vascular complications included in this study for analysis were renal complications (diabetic nephropathy, intracapillary glomerulonephrosis, Kimmelstiel-Wilson syndrome), ophthalmic complications (cataract, retinopathy), neurological complications (amyotrophy, autonomic neuropathy, mononeuropathy, polyneuropathy, autonomic) and peripheral circulatory complications (gangrene, peripheral angiopathy, ulcer), Detail ICD-10 codes for diabetes mellitus and vascular complications used in this study were shown in Additional file 1: Appendix S1. Population data were derived from the WHO Mortality Database and the World Population Prospects 2019 [14, 16].

### Procedural

We identified 135 countries from the database that had included diabetes as a cause of death. Among them, 18 countries used ICD-103 and were therefore excluded. We then extracted mortality data of a 17-year period between 2000 and 2016. Eventually, 108 countries were included for the final analysis (Additional file 1: Appendix S2). Available years of diabetic vascular complication-related deaths, total diabetes-related deaths and mid-year population were provided in Additional file 1: Appendix S3–S5.

### Statistical analysis

To investigate the time trends of vascular complication-related deaths, we first estimated the crude proportions and rates using the number of deaths reporting any of renal, ophthalmic, neurological and peripheral circulatory complications as the numerator, divided by the number of all-cause diabetes-related deaths. Age-standardized proportions and rates were then calculated using mid-year population. The direct standardization method was performed to calculate age-standardized proportions and rates using the age-specific (0–4 years, 5–9 years, 10–14 years, 95–99 years, > 100 years) numbers of diabetic deaths and populations. Secondly, we compared crude and age-standardized proportions and rates of each country, taking the overall estimates of all countries as the reference. Data were reported as odds ratios (ORs). We also compared the differences of the proportions and rates by age groups, regions, DM types, and domestic incomes (based on the World Bank classification) [17].

Using the crude and age-standardized proportions and rates, we performed joinpoint regression to investigate the time trends of diabetic vascular complication-related deaths. This approach has been widely used to study time trends in mortality from causes including infectious [18] and non-infectious diseases. [19] Descriptions of joinpoint regression were shown in Additional file 1: Appendix S6. Briefly, the joinpoint analysis identifies the best fit for inflexion points (“joinpoints”) at which there is a significant change in trends using a series of permutation tests, with Bonferroni adjustment for multiple comparisons. The joinpoint regression is different than other similar models, like piecewise regression because it has the constrain of continuity at the change-point (s) and the choice of the number of joinpoint (s). Single segment trends were expressed as annual percentage changes (APC), while the average annual percentage changes (AAPC) was used to summarize measures of the overall trend. We used the Z test to assess whether an APC or AAPC was significantly different from zero. Joinpoint regression analysis was performed for the overall estimates, and for subgroups by sex, type of complications, regions and domestic income. Lastly, we also used the calendar year 2000 as the reference to compare trends of ORs in different subgroups.

We used Microsoft Excel 2010 for data extraction, sorting, and cleaning. IBM SPSS Statistics (version 25.0) and Joinpoint Regression Program (version 4.7.0.0) were used for data analysis. Results were reported with absolute number, percentage and 95% confidence interval (95% CI). Proportions were reported as per 1000 deaths, and rates were reported in 100,000 person-years. A p value less than 0.05 was considered statistically significant.

## Results

### Overall characteristics

Between 2000 and 2016, a total of 7,108,145 diabetes-related deaths were reported in the 108 selected countries, including 1,904,787 cases (26.8%) that were attributed to vascular complications. By type of diabetes, there were 88,479 cases of T1DM, 687,959 cases of T2DM, 11,466 cases of malnutrition-related DM, 2326 cases of other specified DM and 1,114,557 cases of unspecified DM. Among the 1,904,787 deaths from vascular complications, the largest proportion was attributed to diabetic nephropathy (1,355,085 cases, 71.1%), followed by peripheral circulatory complications (515,293 cases 27.1%), diabetic neuropathy (28,697 cases, 1.5%) and diabetic retinopathy (5751 cases, 0.3%).

### Proportions of deaths

**Fig. 1:**
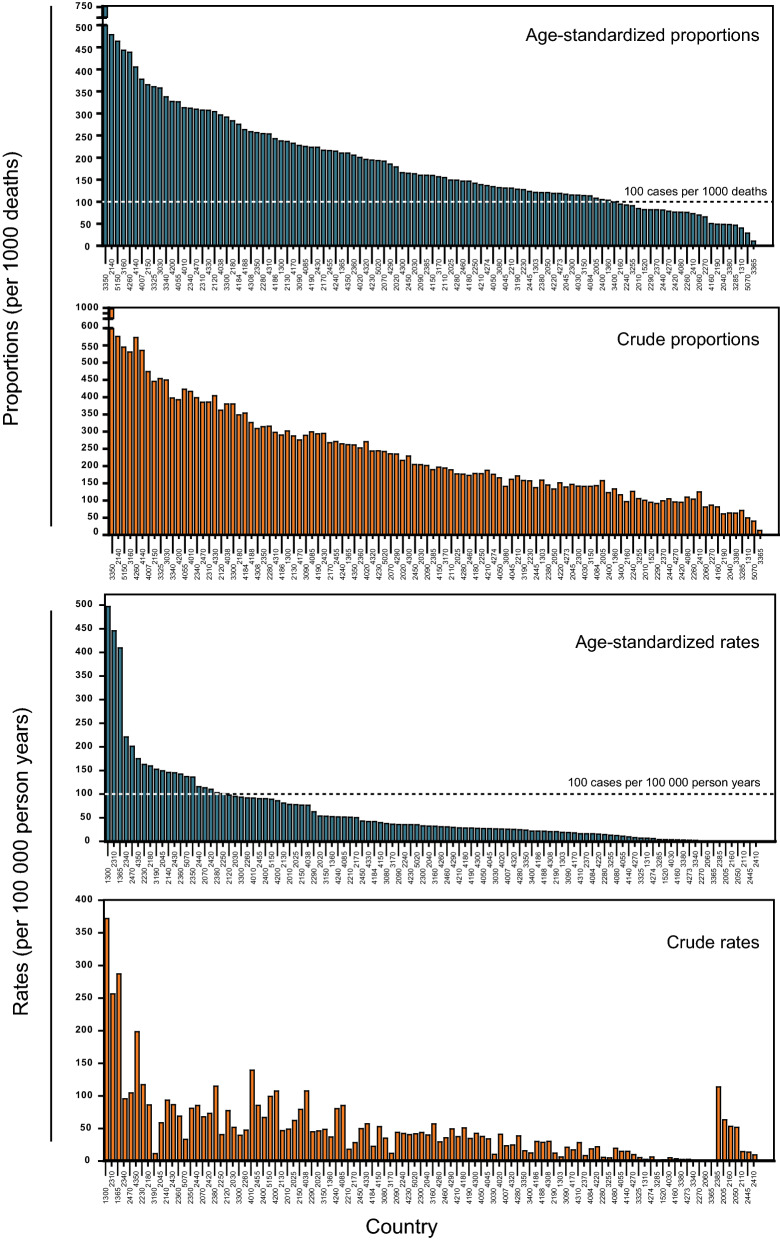
Crude and age-standardized proportions and rates, by country; Data were sorted by the number of age-standardized proportions and rates; dash line denotes 100 cases per 1000 deaths in proportions and 100 cases per 100 000 person years in rates. The number below the X axis was country codes (corresponding country name was shown in Additional file 1: Appendix S17)

Over the 17-year study period, the overall age-standardized proportions of diabetic vascular complication-related deaths were 267.8 (95% CI 267.5–268.1) cases per 1000 deaths. The highest proportion was found in Singapore (759.6 cases per 1000 deaths, 736.8–782.5), while the lowest was in Sri Lanka (10.9 cases per 1000 deaths, 8.7–13.1) (Additional file 1: Appendix S7). Eighty-four countries (77.8%) showed an age-standardized proportion higher than 100 per 1000 deaths. Compared to the overall mean, 68 countries (63.0%) had an OR < 1, while 40 (37.3%) countries had an OR > 1, ranging from 0.05 (0.04–0.06) in Siri Lanka to 3.37 (3.12–3.65) in Singapore (Fig. 1; Additional file 1: Appendix S8). The age-standardized proportions were higher in men (279.6 cases per 1000 deaths, 278.7–279.7) than in women (257.8 cases per 1000 deaths, 257.4–258.3) (Additional file 1: Appendix S9). The proportions of death due to vascular complications were higher in older persons than in younger individuals, the highest age-standardized proportions were found among 45–64 years (311.2 cases per 1000 deaths, 310.5–311.9), while the lowest were found among < 20 years (78.0 cases per 1000 deaths, 73.4–82.6) (Additional file 1: Appendix S10). These differences were consistent in both men and women.

By region, Asia (412.8 cases per 1000 deaths, 411.8–414.5) had higher proportions of death than other regions, followed by North America (287.5 cases per 1000 deaths, 286.9–288.0) and Oceania (262.2 cases per 1000 deaths, 259.3–265.1). The lowest proportions of death were found in Middle East (128 cases per 1000 deaths, 126.5–129.5) and Africa (160.9 cases per 1000 deaths, 158.2–163.8) (Additional file 1: Appendix S11). By type of vascular complications, renal complications had the highest proportions (190.5 cases per 1000 deaths, 190.4–190.9), followed by peripheral circulatory complications (72.5 cases per 1000 deaths, 72.3–72.7), neurological complications (4.0 cases per 1000 deaths, 3.9–4.1) and ophthalmic complications (0.81 cases per 1000 deaths, 0.78–0.83) (Table 1). By domestic income, higher income countries had lower proportions of death (high: 242.3 cases per 1000 deaths, 241.8–242.7; upper-middle: 293.8 cases per 1000 deaths, 293.3–294.3) than lower income countries (lower-middle: 311.5 cases per 1000 deaths, 309.6–313.3; low: 427.9 cases per 1000 deaths, 418.5–437.4).

**Table 1:** Crude and age-standardized proportions and rates from 2000 to 2016, by vascular complication types

Year	Proportions (per 1000 diabetes deaths)				Rates (per 100,000 person years)			
	Neurological	Ophthalmic	Peripheral circulatory	Renal	Neurological	Ophthalmic	Peripheral circulatory	Renal
2000	3.46 (3.43)	0.93 (0.93)	75.64 (77.28)	149.12 (143.47)	0.61 (0.74)	0.16 (0.19)	13.41 (14.91)	26.44 (29.70)
2001	3.51 (3.48)	0.80 (0.80)	82.15 (83.32)	149.17 (145.38)	0.65 (0.77)	0.15 (0.17)	15.23 (15.85)	27.66 (30.69)
2002	2.96 (2.93)	0.75 (0.75)	81.28 (82.27)	146.57 (142.91)	0.59 (0.69)	0.15 (0.16)	16.21 (15.95)	29.23 (32.02)
2003	3.38 (3.36)	0.96 (0.96)	84.70 (84.91)	143.08 (141.96)	0.66 (0.76)	0.19 (0.20)	16.50 (15.35)	27.87 (30.17)
2004	3.38 (3.38)	0.76 (0.76)	81.55 (81.58)	141.06 (140.45)	0.63 (0.72)	0.14 (0.15)	15.12 (13.79)	26.15 (27.98)
2005	3.60 (3.60)	0.74 (0.74)	79.38 (79.34)	142.69 (142.55)	0.68 (0.78)	0.14 (0.15)	15.05 (13.70)	27.05 (28.60)
2006	3.60 (3.60)	0.69 (0.70)	74.94 (75.45)	159.61 (157.82)	0.73 (0.82)	0.14 (0.15)	15.23 (13.13)	32.43 (33.70)
2007	4.85 (4.84)	0.72 (0.72)	74.51 (74.68)	180.51 (179.95)	0.97 (1.07)	0.14 (0.15)	14.96 (12.02)	36.24 (37.09)
2008	5.27 (5.28)	0.75 (0.75)	73.62 (73.19)	183.77 (185.21)	1.02 (1.10)	0.15 (0.15)	14.26 (10.76)	35.59 (35.87)
2009	3.96 (3.96)	0.88 (0.88)	70.80 (70.96)	181.57 (181.09)	0.84 (0.90)	0.19 (0.19)	15.11 (11.35)	38.76 (38.48)
2010	3.91 (3.91)	0.98 (0.98)	69.34 (69.32)	188.77 (189.09)	0.85 (0.89)	0.21 (0.21)	14.99 (11.21)	40.79 (39.91)
2011	3.79 (3.79)	0.84 (0.84)	65.78 (65.67)	219.04 (219.51)	0.83 (0.85)	0.18 (0.18)	14.36 (10.02)	47.81 (45.96)
2012	4.00 (3.99)	0.97 (0.97)	64.23 (63.69)	224.31 (226.22)	0.84 (0.85)	0.20 (0.19)	13.52 (8.82)	47.23 (44.62)
2013	4.67 (4.65)	0.92 (0.92)	66.39 (65.75)	241.43 (243.45)	0.99 (0.98)	0.20 (0.18)	14.03 (8.72)	51.03 (47.40)
2014	4.73 (4.70)	0.64 (0.64)	69.05 (68.50)	237.27 (238.67)	1.01 (0.98)	0.14 (0.12)	14.69 (8.93)	50.46 (46.08)
2015	4.71 (4.69)	0.75 (0.74)	67.18 (66.39)	244.05 (246.44)	1.01 (0.97)	0.16 (0.14)	14.36 (8.64)	52.15 (46.89)
2016	4.30 (4.29)	0.67 (0.67)	61.80 (61.52)	249.17 (250.88)	0.93 (0.88)	0.14 (0.13)	13.33 (7.80)	53.76 (47.45)
Overall	4.04 (4.04)	0.81 (0.81)	72.49 (72.53)	190.64 (190.45)	0.82 (0.87)	0.16 (0.16)	14.71 (11.71)	38.68 (38.08)

Data (crude [age-standardized]) were sorted by year and complication types; proportions (per 1000 diabetes deaths); rates (per 100,000 population years)

### Rates of deaths

Between 2000 and 2016, the overall age-standardized rates of vascular complication related deaths were 53.6 (53.5–53.7) cases per 100,000 person-years (Additional file 1: Appendix S12). The highest rate was found in Mauritius (496.9 cases per 100,000 person-years, 490.1–503.7), while the lowest rate was documented in Siri Lanka (0.45 cases per 100,000 person-years, 0.38–0.52) (Additional file 1: Appendix S7). Eighty-one countries (80.2%) had an age-standardized rate less than 100 cases per 1000 deaths. Compared to the overall mean, 67 countries (66.3%) had an OR < 1, while 34 (33.7%) countries had an OR > 1, ranging from 0.006 (0.005–0.007) in Siri Lanka to 7.15 (6.98–7.32) in Mauritius (Fig. 1; Additional file 1: Appendix S8). Vascular complication related death rates were higher in older persons than for their younger counterparts. The highest rate was observed in the age group over 65 years (325.81 cases per 100,000 person-years, 325.4–326.3), while the lowest was found in persons of age < 20 years (0.10 cases per 100,000 person-years, 0.09–0.11) (Additional file 1: Appendix S13).

By region, North America had the highest age-standardized death rate due to vascular complications (94.7 cases per 100,000 person-years, 94.5–95.0), followed by South America (66.0 cases per 100,000 person-years, 65.7–66.2) and Oceania (50.8 cases per 100,000 person-years, 50.1–51.4). By contrast, the Middle East (11.2 cases per 100,000 person-years, 11.1–11.4) and Africa (13.0 cases per 100,000 person-years, 12.7–13.2) had the lowest rates (Additional file 1: Appendix S14). By complication type, the highest rate was found in renal complications (38.1 cases per 100,000 person-years, 38.0–38.2), followed by peripheral circulatory complications (11.7 cases per 100,000 person-years, 11.6–11.8), neurological complications (0.87 cases per 100,000 person-years, 0.86–0.88), and ophthalmic complications (0.16 cases per 100,000 person-years, 0.15–0.17) (Table 1). Higher age-standardized death rates were observed in higher income countries (high: 46.6 cases per 100,000 person-years, 46.5–46.7; upper-middle: 75.7 cases per 100,000 person-years, 75.6–75.8) than in the other groups (lower-middle: 27.6 cases per 100,000 person-years, 27.4–27.8; low: 12.9 cases per 100,000 person-years, 12.5–13.3).

### Trend of diabetic death rates due to vascular complications

**Fig. 2:**
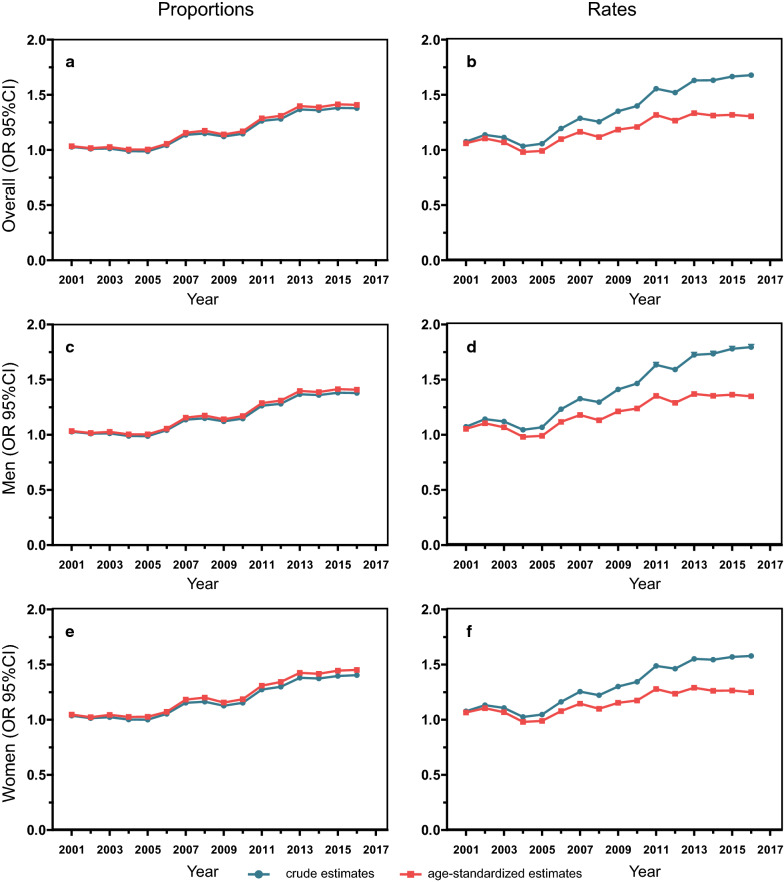
Odds ratios (ORs) of crude and age-standardized proportions and rates compared to year 2000, by sex; Data presented as estimate ± 95% CI; Circles, crude estimates; Squares, age-standardized estimates; **a**, **c**, **e** represent proportions; **b**, **d**, **f** represent rates; **a**, **b** were ORs in overall estimates; **c**, **d** were ORs in male estimates; **e**, **f** were ORs in female estimates

**Fig. 3:**
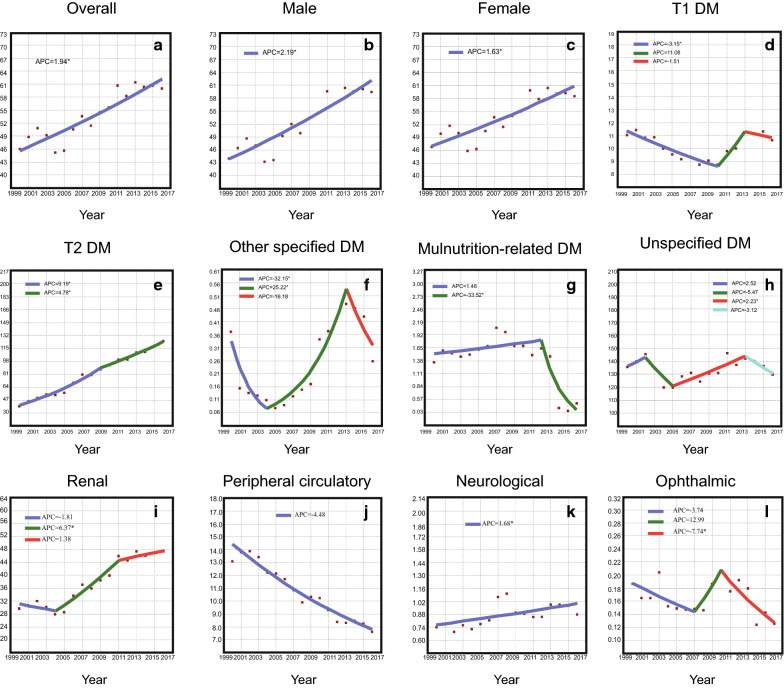
Trend of age-standardized mortality rates (per 100 000 person years) in overall and subgroups by sex, diabetes types and complication types; red squares denote the observed values and line denotes the slope of the APC in different segments; A for overall, B and C for subgroups by sex (B: male, C: female); D-H for subgroups by diabetes types (D: T1DM; E: T2DM; F: other specified DM; G: malnutrition-related DM; H: unspecified DM); I-L for subgroups by complication types (I: renal complication; J: peripheral circulatory complication; K: neurological complication; L: ophthalmic complication); APC = annual percentage change; * denotes a *p* value less than 0.05

**Fig. 4:**
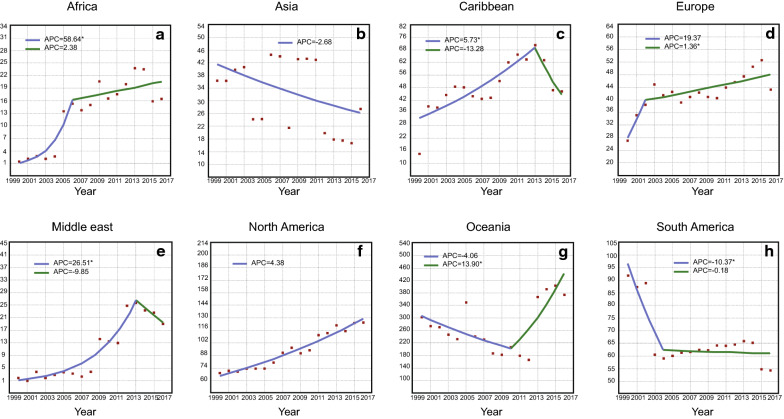
Trend of age-standardized mortality rates (per 100 000 person years) in subgroups by regions; red squares denote the observed values and line denotes the slope of the APC in different segments; A for Africa, B for Asia; C for Caribbean; D for Europe; E for Middle East; F for North America; G for Oceania; H for South America; D-H for subgroups by diabetes types (D: T1DM; E: T2DM; F: other specified DM; APC = annual percentage change; * denotes a *p* value less than 0.05

From 2000 to 2016, overall rates of deaths due to diabetes vascular complications had increased 30.8%, from 46.0 cases per 100,000 person-years (45.7–46.3) to 60.2 cases (59.7–60.3) per 100,000 person-years. Compared to the rates in the year 2000, the age-standardized OR increased from 1.06 (1.05–1.07) in 2001 to 1.30 (1.29–1.31) in 2016, with highest OR occurred in 2013 (1.33, 1.32–1.35) (Fig. 2). The trend of OR also varied by different subgroups (Additional file 1: Appendix S15, S16). The joinpoint model analysis showed that the overall AAPC of rate was 1.9% (p < 0.05, 1.4%–2.4%). Both men (AAPC = 2.2%; 1.7%–2.7%; p < 0.05) and women (AAPC = 1.6%; 1.1%–2.1%; p < 0.05) showed an upward trend during the study period. However, the trend varied across different subgroups. As shown in Table 2 and Fig. 3, By DM types, the AAPC was increased in patients with T2DM (AAPC = 7.2%; 6.3%–8.2%; p < 0.05) but decreased in persons with T1DM (AAPC = − 8.7%; − 14 to − 3.1%; p < 0.05). Similarly, by complication types, the AAPC was higher for diabetic nephropathy (AAPC = 2.7%; 1.2% to 4.3%; p < 0.05) and diabetic neuropathy (AAPC = 1.7%; 0.4–2.9%; p < 0.05), but lower for diabetic peripheral circulatory complications (AAPC = − 4.5%; − 5.0 to − 3.9%; p < 0.05). By regions, as shown in Fig. 4, trend of annual rate increased in Africa, Caribbean, Europe, Middle East, North America and Oceania, but decreased in Asia and South America.

**Table 2:** Crude and age-standardized average annual percentage change in rates by overall and different subgroups

Subgroup trend	Crude rates				Age-standardized rates			
	AAPC	95% CI		*P* *	AAPC	95% CI		*P* *
Total	3.6*	3.1	4.1	< 0.05	1.9*	1.4	2.4	< 0.05
By sex								
Male	4.1*	3.5	4.6	< 0.05	2.2*	1.7	2.7	< 0.05
Female	3.2*	2.6	3.7	< 0.05	1.6*	1.1	2.1	< 0.05
By DM type								
T1DM	0.8	− 1.4	3	0.7	− 0.3	− 2.5	1.9	− 0.3
T2DM	7.2*	6.3	8.2	14.9	7.2*	6.3	8.2	14.9
Malnutrition-related DM	− 7.6*	− 12.8	− 2	− 2.6	− 8.7*	− 14	− 3.1	− 3
Other specified DM	1.3	− 5.9	9.1	0.4	− 0.4	− 7.3	7.1	− 0.1
Unspecified DM	1.1	− 1.8	4	0.7	− 0.2	− 3	2.6	− 0.2
By complication								
Neurological	3.2*	1.9	4.5	< 0.05	1.7*	0.4	2.9	< 0.05
Ophthalmic	− 0.8	− 7.2	6.1	0.8	− 2.4	− 8.6	4.3	0.5
Peripheral circulatory	< 0.05	− 1.3	1.4	1	− 4.5*	− 5	− 3.9	< 0.05
Renal	4.3*	2.7	5.9	< 0.05	2.7*	1.2	4.3	< 0.05
By region								
Africa	23.4*	12.2	35.8	< 0.05	20.7*	10.4	31.8	< 0.05
Asia	− 0.6	− 4.1	3	0.7	− 2.9	− 6.2	0.6	0.1
Caribbean	3.6	− 0.4	7.8	0.1	1.9	− 2.4	6.4	0.4
Europe	4.7*	2.6	6.8	< 0.05	3.5*	0.2	6.8	< 0.05
Middle east	21.1*	13.2	29.6	< 0.05	18.7*	10.8	27.2	< 0.05

AAPC means average annual percentage change, and the parametric method was used to calculate 95% CI; / denotes no available data

*p value < 0.05

## Discussions

### Main findings

In this study, we reported on the burden of vascular complications in a total of over 7 million diabetic deaths in 108 countries during 2000–2016. The proportions and rates of deaths due to vascular complications varied substantially by DM types, complications and geographical areas. Overall, the trend of diabetic mortality due to vascular complications continued to increase from 2000 through 2016.

### Increased mortality rate in vascular complications

Over the last two decades, prevalence of diabetes increased rapidly despite promoted programs of prevention and intervention, [1] As a consequence, the prevalence of diabetic complications may also have changed accordingly. [20] Edward and colleagues demonstrated a downward trend of diabetes-related complications between 1990 and 2010, including end stage renal disease (ESRD) and lower extremity amputation (LEA), which had declined by 29% and 53%, respectively. [4] Harding et al. summarized the evidence and concluded that, [21] with regard to vascular complications, incidences of LEA [22, 23], diabetic retinopathy [24] and ESRD [4, 25] decreased, while diabetic neuropathy increased during the last few decades. Given these findings, the mortality rate due to diabetic vascular complications seemed to have declined during the same period. However, in this study, we found that the overall trend of diabetes-related deaths due to vascular complications continued to increase between 2000 and 2016. Specifically, an upward trend was observed in renal and neurological complication-related deaths, while a downward trend was observed in peripheral circulatory and ophthalmic complication-related deaths. Additionally, we found that the mortality trend due to vascular complications in T1DM decreased during 2000–2010 but increased during 2010–2013, yet the turning points were not present in the combined analysis. On the other hand, for T2DM, the upward trend of mortality was persistent during 2000–2016. These findings indicated that hard endpoints related to vascular complications in T2DM present a serious challenge for diabetes care and management.

### Increased mortality in diabetic nephropathy

In this study, we observed that the mortality trends varied by type of vascular complications. The overall proportions and rates of deaths due to vascular complications had increased 43.8% and 30.4%, respectively. However, this increase was primarily driven by growth in the renal complications, which had increased 74.8% and 59.8%, respectively, during the study period. This finding stressed the fact that diabetic nephropathy remains a top priority in the prevention and treatment of diabetes complications. The increased trend of diabetic nephropathy related deaths may partly be explained by the growing prevalence of diabetes. Approximately 40% of patients with diabetes would develop kidney disease. [26] Worldwide, it is estimated that 80% of ESRD cases are caused by diabetes or hypertension. [21] Diabetes is the leading cause of ESRD in the United States and the 5-year survival for patients with ESRD is less than 40%. [27, 28] Additionally, diabetic nephropathy was diagnosed using albuminuria historically. Over the past two decades, non-albuminuric reductions in eGFR are increasingly recognized as diabetic nephropathy. Previously ESRD mortality might have been attributed to hypertension but more recently might have been attributed to diabetic nephropathy. [21, 29] Therefore, the reported increase in diabetic nephropathy related mortality might be affected by a change in understanding of diabetic nephropathy.

Of note, intensive glycemic control has been widely studied for its benefits in renal outcomes [30]. In a meta-analysis of clinical trials (ACCORD, ADVANCE, UKPDS, and VADT), Zoungas and colleagues found reduced kidney events with intensive glucose control [2]. Another study showed consistent reductions in microalbuminuria surrogates [31]. However, these outcomes do not necessarily translate to downstream improvements in their corresponding hard endpoints [32, 33]. In contrast, almost all studies have consistently shown a significant increase in severe hypoglycemia and related mortality [34–37]. Correspondingly, CKD is closely correlated with hypoglycemia and may increase the risks of hypoglycemia-induced death and cardiovascular events [38, 39]. Hence, the pros and cons of intensive glycemic control for patients with diabetic nephropathy should be carefully balanced [42].

### Differences of mortality rate by geography and time scales

This study also demonstrated differences in mortality trends by countries, geographic areas, domestic incomes and time scales. These findings could be explained by pathophysiological differences among individuals of diverse ethnicity, cultural background or variations in diabetes control. Bullock et al. demonstrated that, between 2000 and 2013, the incidence of ESRD with diabetes for American Indian/Alaska Native had declined by 28%, followed by Hispanic (22%), non-Hispanic white (14%) and non-Hispanic black people (13%) in the United States. Yet, during the same period, ESRD incidence remained relatively stable in Asian individuals with diabetes [25]. Additionally, income may also contribute to variable rates of death. A study conducted in England showed that risks of inpatient admission for diabetes increases with socioeconomic deprivation. [40] Reports from IDF have shown that availability of diabetic drugs differs tremendously across income groups. The availability of diabetes supplies ranged from ~ 80% in high-income countries to < 15% in low-income countries. [41] Last, the shifting diagnosis criteria for disease may account for the turning points in some regions. For example, in Australia, a falling trend of diabetic nephropathy was observed between 2000 and 2012 (230 diabetic nephropathy cases in 2012, 21.3%), whereas this trend was sharply increase from 2013 to 2016 (1240 diabetic nephropathy cases in 2013, 65.9%). These internal and external inequalities might help explain the differences in the proportions and rates of deaths by regions and countries.

### Co-existing microvascular and macrovascular complications

The increasing trend of deaths from microvascular complications may correlate with the rising trend of deaths from macrovascular complications [42, 43]. Vascular impairment is closely associated with diabetes. On the one hand, T2DM has a substantial effect on micro- and macrovascular disease and all-cause mortality rates in all age groups, having more pronounced impact on younger individuals [44]. On the other hand, vascular dysfunction may also contribute to the development of T2DM [45]. In patients with T2DM, carotid atherosclerosis parameters predict both cardiovascular and renal outcomes leading to an improved renal risk stratification [46]. An elevated triglyceride-glucose index (TyG index), which is a surrogate of vascular impairment, has been shown positively associated with diabetes prognosis [47] and nephric microvascular damage [48]. Co-existing microvascular and macrovascular complications are frequently observed in patients with diabetes. In this regard, there are considerable difficulties in issuing a death certificate by differentiating microvascular complication from macrovascular complications. The presence and severity of microvascular complications contribute independently to increase the risk of all-cause mortality and cardiovascular events [49]. Therefore, microangiopathy and macroangiopathy may no longer be considered as entirely separate entities but a continuum of systemic vascular damage [50].

### Limitations of the study

To our knowledge, this was the first study to evaluate the mortality trends of diabetic vascular complications in the global landscape. However, there were several limitations. First, we used the WHO mortality database, in which data of some countries with a significant diabetic population, including such as China and India, where accumulate the largest number of people with diabetes mellitus worldwide, are not available. Therefore, the mortality trends of those countries were not reflected in our estimation. Nevertheless, our methodology can be applied to other country-level data for a better understanding of the mortality trend of diabetic vascular complications. Second, the ICD codes used by different countries to identify underlying cause of death may also generate potential bias. In this study, the highest overall age-standardized proportion of diabetic vascular complication-related death was found in Singapore (759.6 cases per 1000 deaths, 95% CI 736.8–782.5), while the lowest was in Sri Lanka (10.9 cases per 1000 deaths, 95% CI 8.7–13.1). The difference in mortality might result from the assigned code as unspecified diabetes mellitus without complications (E14.9). The percentage of E14.9-coded deaths was 1.48% (16/1664) in Singapore but 97.64% (8336/8535) in Sri Lanka. Additionally, patients with diabetes, especially the elderly, often have several complications including hypoglycemia, cardiovascular disease and stroke. Deaths from these complications may be the result of vascular damage but recorded as other causes in different countries. Moreover, LEA is often used as a neurological end point based on the observation that many amputations are a result of diabetes neuropathy and ulcers/infections in the insensate foot. But this is not recorded as a neuropathy end point basing on the ICD rules. Therefore, the comparability and representatives of mortality might be affected across different countries even though the data available from the WBD comprise deaths registered in national vital registration systems and underlying cause of death be coded by the relevant national authority. Third, since certain years of data in some countries were missing, we were not able to analyze trends by country, but estimated the mortality trend with aggregated regional data. Last, estimates of proportions and rates were not based on diabetes prevalence over time as such data were not available. Instead, we used total diabetes deaths, which was indicative of the burden of diabetes.

## Conclusions

In conclusion, we have demonstrated an upward trend of diabetic vascular complications related deaths during the period of 2000–2016. This rising trend of deaths was mainly the result of renal complication in T2DM. Although morbidity of diabetic vascular complications had declined in the past few decades, mortality had continued to climb. This finding indicates an urgent need for developing and implementing effective strategies to reduce the burden of diabetic vascular complications particularly renal damages.

## Supplementary information

**Additional file 1.** **Appendix S1**: Details of ICD codes for diabetes mellitus and for diabetic vascular complications used in this study. **Appendix S2**: Flow chart of countries selection. **Appendix S3**: Available years (grey) for diabetes microvascular complication deaths, by country. **Appendix S4**: Available years (grey) for total diabetes deaths, by country. **Appendix S5**: Available years (grey) for midyear population, by country. **Appendix S6**: Description of joinpoint regression model. **Appendix S7**: Crude and age-standardized proportions and rates of diabetes microvascular complication related deaths, by country. **Appendix S8**: Crude and age-standardized odd ratios of proportions and rates compared to overall, by country. **Appendix S9**: Crude and age-standardized proportions from 2000 to 2016, by sex. **Appendix S10**: Crude age specific proportions from 2000 to 2016, by sex. **Appendix S11**: Crude and age-standardized proportions from 2000 to 2016, by region. **Appendix S12**: Crude and age-standardized rates from 2000 to 2016, by sex. **Appendix S13**: Crude age specific rates from 2000 to 2016, by sex. **Appendix S14**: Crude and age-standardized rates from 2000 to 2016, by region. **Appendix S15**: Age-standardized odd ratio of rates compared to year 2000, by different subgroups. **Appendix S16**: Age-standardized odd ratio of proportions compared to year 2000, by different subgroups. **Appendix S17**: Country code and corresponding country name.

## Data Availability

Data of this article were available from the WHO mortality database: https://www.who.int/healthinfo/statistics/mortality_rawdata/en/.

## Abbreviations

T2DM: Type 2 diabetes mellitus
WHO: World Health Organization
ICD: International Classification of Diseases
APC: Annual percentage changes
AAPC: Average annual percentage changes
SPSS: Statistical Product and Service Solutions
CI: Confidence interval
ESRD: End stage renal disease
CKD: Chronic kidney disease
LEA: Lower extremity amputation
TyG: Triglyceride-glucose
