# Supplementary material for: Global trend of diabetes mortality attributed to vascular complications, 2000–2016
Source: Cardiovasc Diabetol. 2020 Oct 20;19:182. doi: 10.1186/s12933-020-01159-5 (PMC7573870; doi:10.1186/s12933-020-01159-5)
Supplement: Supplementary file 1 — Additional file 1. Appendix S1: Details of ICD codes for diabetes mellitus and for diabetic vascular complications used in this study. Appendix S2: Flow chart of countries selection. Appendix S3: Available years (grey) for diabetes microvascular complication deaths, by country. Appendix S4: Available years (grey) for total diabetes deaths, by country. Appendix S5: Available years (grey) for midyear population, by country. Appendix S6: Description of joinpoint regression model. Appendix S7: Crude and age-standardized proportions and rates of diabetes microvascular complication related deaths, by country. Appendix S8: Crude and age-standardized odd ratios of proportions and rates compared to overall, by country. Appendix S9: Crude and age-standardized proportions from 2000 to 2016, by sex. Appendix S10: Crude age specific proportions from 2000 to 2016, by sex. Appendix S11: Crude and age-standardized proportions from 2000 to 2016, by region. Appendix S12: Crude and age-standardized rates from 2000 to 2016, by sex. Appendix S13: Crude age specific rates from 2000 to 2016, by sex. Appendix S14: Crude and age-standardized rates from 2000 to 2016, by region. Appendix S15: Age-standardized odd ratio of rates compared to year 2000, by different subgroups. Appendix S16: Age-standardized odd ratio of proportions compared to year 2000, by different subgroups. Appendix S17: Country code and corresponding country name. [file 12933_2020_1159_MOESM1_ESM.pdf]

Additional file 1

Global Trend of Diabetes Mortality Attributed to Vascular Complications, 2000 - 2016

LING Wei, MD; HUANG Yi, MD; HUANG Yan-Mei, MD; FAN Rong-Rong, MD; SUI Yi, MD; ZHAO Hai-Lu, PhD

Appendix S1. Details of ICD codes for diabetes mellitus and for diabetic vascular complications used in this study

Appendix S2. Flow chart of countries selection

Appendix S3. Available years (grey) for diabetes microvascular complication deaths, by country

Appendix S4. Available years (grey) for total diabetes deaths, by country

Appendix S5. Available years (grey) for midyear population, by country

Appendix S6. Description of joinpoint regression model

Appendix S7. Crude and age-standardized proportions and rates of diabetes microvascular complication related deaths, by country

Appendix S8. Crude and age-standardized odd ratios of proportions and rates compared to overall, by country

Appendix S9. Crude and age-standardized proportions from 2000 to 2016, by sex

Appendix S10. Crude age specific proportions from 2000 to 2016, by sex

Appendix S11. Crude and age-standardized proportions from 2000 to 2016, by region

Appendix S12. Crude and age-standardized rates from 2000 to 2016, by sex

Appendix S13. Crude age specific rates from 2000 to 2016, by sex

Appendix S14. Crude and age-standardized rates from 2000 to 2016, by region

Appendix S15. Age-standardized odd ratio of rates compared to year 2000, by different subgroups

Appendix S16. Age-standardized odd ratio of proportions compared to year 2000, by different subgroups

Appendix S17. Country code and corresponding country name

Appendix S1: Details of ICD codes for diabetes mellitus and for diabetic vascular complications used in this study

Diabetes mellitus (E10-E14)

E10: Type 1 diabetes mellitus

[See after E14 for subdivisions]

Incl.:

diabetes (mellitus):

- brittle
- juvenile-onset
- ketosis-prone

Excl.:

diabetes mellitus (in):

- malnutrition-related
- neonatal
- pregnancy, childbirth and the puerperium

glycosuria:

- NOS
- renal

impaired glucose tolerance
postsurgical hypoinsulinaemia

E11Type 2 diabetes mellitus

[See after E14 for subdivisions]

Incl.:

diabetes (mellitus)(nonobese)(obese):

- adult-onset
- maturity-onset
- nonketotic
- stable

non-insulin-dependent diabetes of the young

Excl.:

diabetes mellitus (in):

- malnutrition-related
- neonatal
- pregnancy, childbirth and the puerperium

glycosuria:

- NOS
- renal

impaired glucose tolerance
postsurgical hypoinsulinaemia

E12 Malnutrition-related diabetes mellitus

[See after E14 for subdivisions]

Incl.:

malnutrition-related diabetes mellitus:

- type 1
- type 2

Excl.:

diabetes mellitus in pregnancy, childbirth and the puerperium
glycosuria:

- NOS
- renal (E74.8)

impaired glucose tolerance
neonatal diabetes mellitus
postsurgical hypoinsulinaemia

E13 Other specified diabetes mellitus

[See after E14 for subdivisions]

Excl.:

diabetes mellitus (in):

- malnutrition-related
- neonatal

- pregnancy, childbirth and the puerperium
- type 1
- type 2

glycosuria:

- NOS
- renal

impaired glucose tolerance
postsurgical hypoinsulinaemia

E14 Unspecified diabetes mellitus

[See after E14 for subdivisions]

Incl.:
diabetes NOS
Excl.:
diabetes mellitus (in):

- malnutrition-related
- neonatal
- pregnancy, childbirth and the puerperium
- Type 1
- Type 2

glycosuria:

- NOS
- renal

impaired glucose tolerance
postsurgical hypoinsulinaemia

The following subdivisions are for use with categories E10-E14

.0 With coma

Diabetic:

- coma with or without ketoacidosis
- hyperosmolar coma
- hypoglycaemic coma

Hyperglycaemic coma NOS

.1 With ketoacidosis

Diabetic:	
acidosis	without mention of coma
ketoacidosis	

.2† With renal complications

Diabetic nephropathy
Intracapillary glomerulonephrosis
Kimmelstiel-Wilson syndrome

.3† With ophthalmic complications

Diabetic:

- cataract
- retinopathy

.4† With neurological complications

Diabetic:

- amyotrophy
- autonomic neuropathy
- mononeuropathy
- polyneuropathy
- autonomic

.5† With peripheral circulatory complications

Diabetic:

- gangrene
- peripheral angiopathy
- ulcer

.6 With other specified complications

Diabetic arthropathy

Neuropathic diabetic arthropathy

.7 With multiple complications

.8 With unspecified complications

.9 Without complications

---

† denotes codes of diabetic complications used in this study

T**ype 1 DM** with renal complications (E10.2), ophthalmic complications (E10.3), neurological complications (E10.4) and peripheral circulatory complications (E10.5); **Type 2 DM** with renal complications (E11.2), ophthalmic complications (E11.3), neurological complications (E11.4) and peripheral circulatory complications (E11.5); **Malnutrition-related DM** with renal complications (E12.2), ophthalmic complications (E12.3), neurological complications (E12.4) and peripheral circulatory complications (E12.5); **Other specified DM** with renal complications (E13.2), ophthalmic complications (E13.3), neurological complications (E13.4) and peripheral circulatory complications (E13.5); **Unspecified DM** with renal complications (E14.2), ophthalmic complications (E14.3), neurological complications (E14.4) and peripheral circulatory complications (E14.5).

Appendix S2. Flow-chart of countries selection

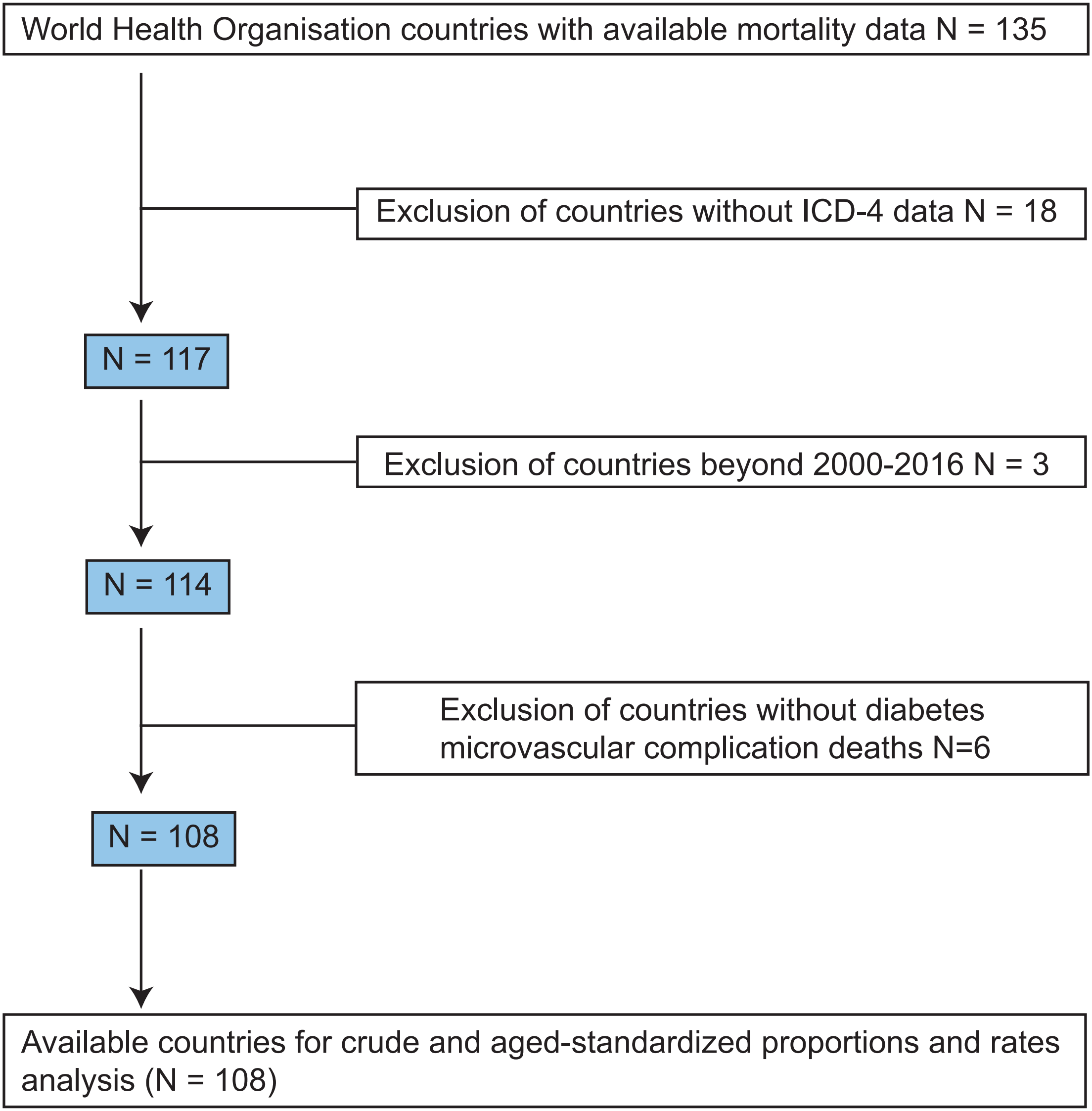

Appendix S3. Available years (grey) for diabetes microvascular complication deaths, by country

Code	2000		2002		2004		2006		2008		2010		2012		2014		2016
1300						■	■	■	■	■	■	■	■	■	■	■	■
1303													■	■	■	■	■
1310	■	■	■	■	■			■	■	■	■	■	■	■	■		
1360		■	■	■	■	■	■	■	■	■	■	■	■	■	■	■	■
1365						■	■	■	■	■	■	■	■	■	■	■	■
1520										■				■			
2005					■		■			■	■	■	■	■	■	■	■
2010	■		■		■	■	■	■	■	■			■	■	■	■	■
2020	■	■	■	■	■	■	■	■	■	■	■	■	■	■	■	■	■
2025						■	■	■	■	■	■	■	■	■	■	■	■
2030	■	■	■	■	■	■	■	■	■	■	■	■	■	■	■		
2040			■	■	■	■	■	■	■	■	■	■	■	■			
2045								■	■	■	■	■	■	■	■	■	■
2050		■	■	■	■	■	■	■	■	■	■	■	■	■	■	■	■
2060	■	■	■	■													
2070	■	■	■	■	■	■	■	■	■	■	■	■	■	■	■	■	■
2090	■	■	■	■	■	■	■	■	■	■	■	■	■	■	■	■	■
2110				■	■	■	■	■	■					■			
2120	■	■	■	■	■	■	■	■	■	■	■	■	■	■	■	■	■
2130	■	■	■	■	■	■	■	■	■	■	■	■	■	■	■	■	■
2140	■	■	■	■	■	■	■	■	■	■	■	■	■	■	■	■	■
2150		■	■	■	■	■	■	■	■	■	■	■	■	■	■	■	■
2160		■	■	■	■	■	■	■	■	■	■	■	■	■	■	■	
2170	■	■	■	■	■	■	■	■	■	■	■	■	■	■			
2180	■	■	■	■	■	■	■	■	■	■	■	■	■	■	■	■	■
2190	■	■	■	■	■	■	■	■	■	■	■	■	■	■	■	■	
2210			■	■	■	■	■	■	■	■	■	■	■	■	■	■	■
2230			■	■	■	■	■	■	■	■	■	■	■	■	■	■	■
2240	■	■	■	■	■	■	■	■	■	■	■	■	■	■	■	■	
2250						■	■	■	■	■	■	■	■	■	■	■	■
2260						■				■	■	■	■	■	■		
2270		■	■	■	■												
2280									■	■	■	■	■	■	■		
2290		■	■	■	■	■	■	■	■	■	■	■	■	■	■		
2300	■	■	■	■	■	■	■	■	■	■	■	■	■	■	■	■	■
2310	■	■	■	■	■	■	■	■	■	■	■	■	■	■	■	■	■
2340	■	■	■	■	■	■	■	■	■	■	■	■	■	■	■	■	■
2350	■	■	■	■	■	■	■	■	■	■	■	■	■	■	■	■	■
2360	■	■	■	■	■	■	■	■	■	■	■	■	■	■	■	■	■
2370	■	■	■	■	■	■	■	■	■	■	■	■	■	■	■	■	■
2380	■	■	■	■	■	■	■	■	■	■	■	■	■	■	■	■	■
2385	■		■	■		■		■	■	■	■	■	■	■	■	■	■
2400				■		■	■		■	■	■	■	■	■	■	■	
2410									■								
2420				■	■	■	■	■	■	■	■	■	■	■	■	■	■
2430	■	■	■	■	■	■	■	■	■	■	■	■	■	■	■		
2440	■	■	■	■	■	■	■	■	■	■	■	■	■				
2445							■		■					■	■	■	
2450	■	■	■	■	■	■	■	■	■	■	■	■	■	■	■	■	■
2455	■	■	■	■	■	■	■	■	■	■	■	■				■	■
2460	■	■	■	■	■	■	■	■	■	■	■	■	■	■	■	■	■
2470	■	■	■	■	■	■	■	■	■	■	■	■	■	■	■		
3030																	■
3080					■	■	■	■	■	■	■	■	■	■	■		
3090		■	■	■	■	■	■	■	■	■	■	■	■	■	■	■	■
3150	■	■	■	■	■	■	■	■	■	■	■	■	■	■	■	■	■
3160	■	■	■	■	■	■	■	■	■	■	■	■	■	■	■	■	■
3170									■	■	■	■	■			■	
3190	■	■	■	■	■	■	■	■	■	■	■	■	■	■	■	■	■
3255								■			■	■	■	■	■	■	■
3285											■				■		■
3300	■	■	■	■			■	■		■	■	■					
3325																	■
3340										■							
3350													■	■	■	■	■
3365							■										
3380	■		■	■	■	■	■										
3400										■	■	■	■	■	■	■	■
4007								■									■
4010			■	■	■	■	■	■	■	■	■	■	■	■	■	■	■
4020	■	■	■	■	■	■	■	■	■	■	■	■	■	■	■	■	■
4030														■	■	■	
4038	■	■	■	■	■	■	■	■	■	■	■	■	■	■	■	■	■
4045	■	■	■	■	■	■	■	■	■	■	■	■	■	■	■	■	■
4050	■	■	■	■	■	■	■	■	■	■	■	■	■	■	■	■	
4055										■	■	■	■		■		
4080	■	■	■	■	■	■	■	■	■	■	■	■	■	■	■	■	■
4084					■	■	■	■	■	■	■	■	■	■	■	■	■
4085	■	■	■	■	■	■	■	■	■	■	■	■	■	■	■	■	■
4140															■	■	■
4150	■	■	■	■	■	■	■	■	■	■	■	■	■	■	■	■	■
4160		■	■	■	■			■	■	■							
4170								■	■	■	■	■	■	■	■	■	
4180		■	■	■	■	■	■	■	■	■	■	■	■	■	■	■	■
4184	■	■	■	■	■	■	■	■	■	■	■	■	■	■	■	■	■
4186									■	■	■	■	■	■	■	■	
4188		■	■	■	■	■	■	■	■	■	■	■	■	■	■	■	■
4190	■	■	■	■	■	■	■	■	■	■	■	■	■	■	■	■	■
4200	■	■	■	■	■	■	■	■	■	■	■	■	■	■	■	■	■
4210	■	■	■	■	■	■	■	■	■	■	■	■	■	■	■	■	■
4220	■	■	■	■	■	■	■	■	■	■	■	■	■	■	■	■	■
4230	■	■	■	■	■	■	■	■	■	■	■	■	■	■	■	■	■
4240			■	■				■	■	■	■	■	■	■	■	■	■
4260								■	■	■	■	■	■	■	■	■	■
4270	■	■	■	■	■	■	■	■	■	■	■	■	■	■	■	■	■
4273																■	
4274											■		■	■	■		
4280	■	■	■	■	■	■	■	■	■	■	■	■	■	■	■	■	■
4290	■	■	■	■	■	■	■	■	■	■	■	■	■	■	■	■	■
4300	■	■	■	■	■	■	■	■	■	■	■	■	■	■	■	■	■
4308		■	■	■	■	■	■	■	■	■	■	■	■	■	■	■	■
4310		■	■	■	■	■	■	■	■	■	■	■	■	■	■	■	■
4320		■	■	■	■	■	■	■	■	■	■	■	■	■	■	■	■
4330	■	■	■	■	■	■	■	■	■	■	■	■	■	■	■	■	■
4350	■	■	■														
5020	■	■	■	■	■	■	■	■	■	■	■	■	■	■	■	■	■
5070		■	■	■	■	■	■	■	■	■	■	■	■				
5150	■	■	■	■	■	■	■	■	■	■	■	■	■	■	■	■	

Data are sorted by country code, grey cells denote available data and white cells denote unavailable data

Appendix S4. Available years (grey) for total diabetes deaths, by country

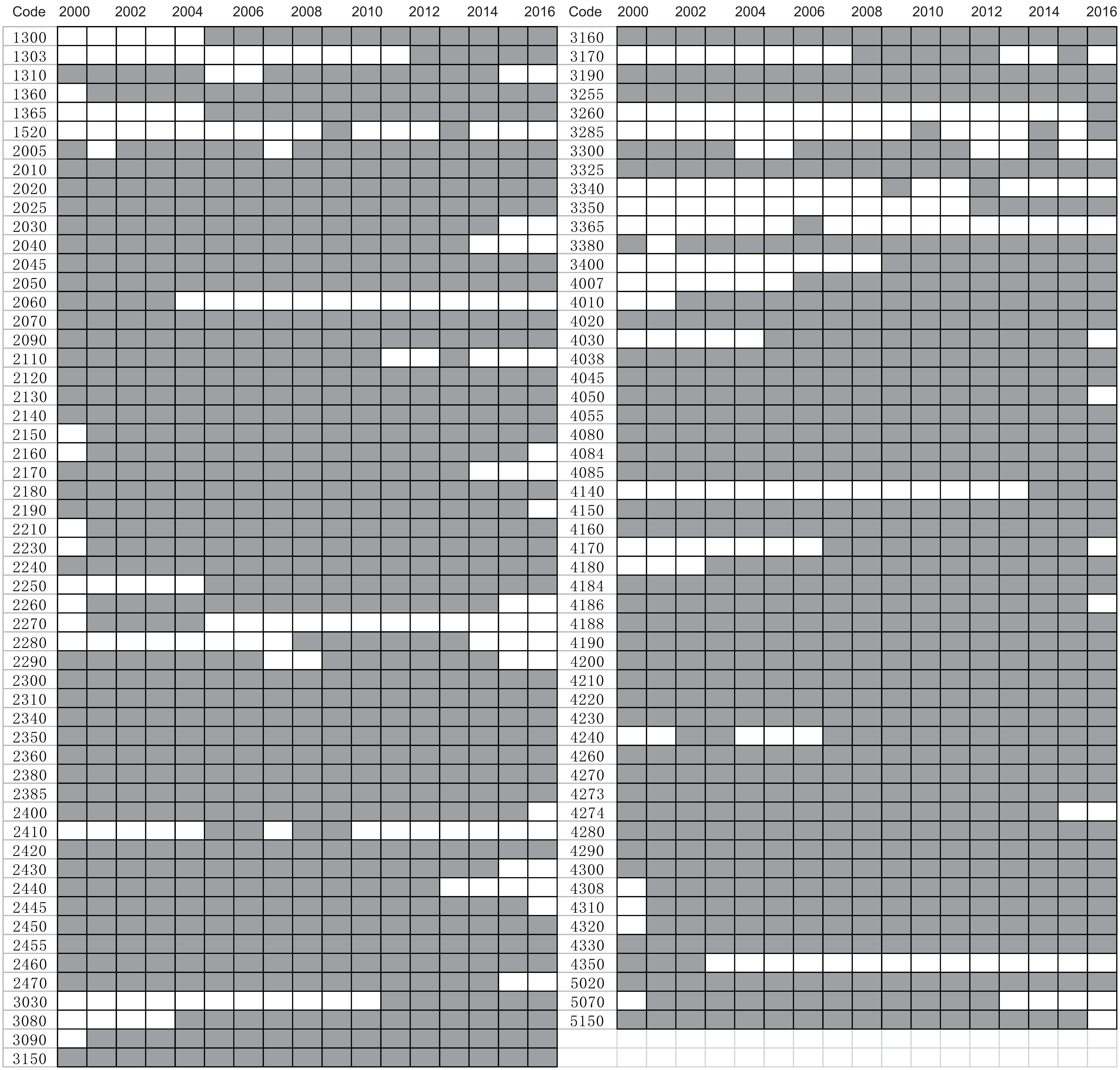

Data are sorted by country code, grey cells denote available data and white cells denote unavailable data

Appendix S5. Available years (grey) for midyear population, by country

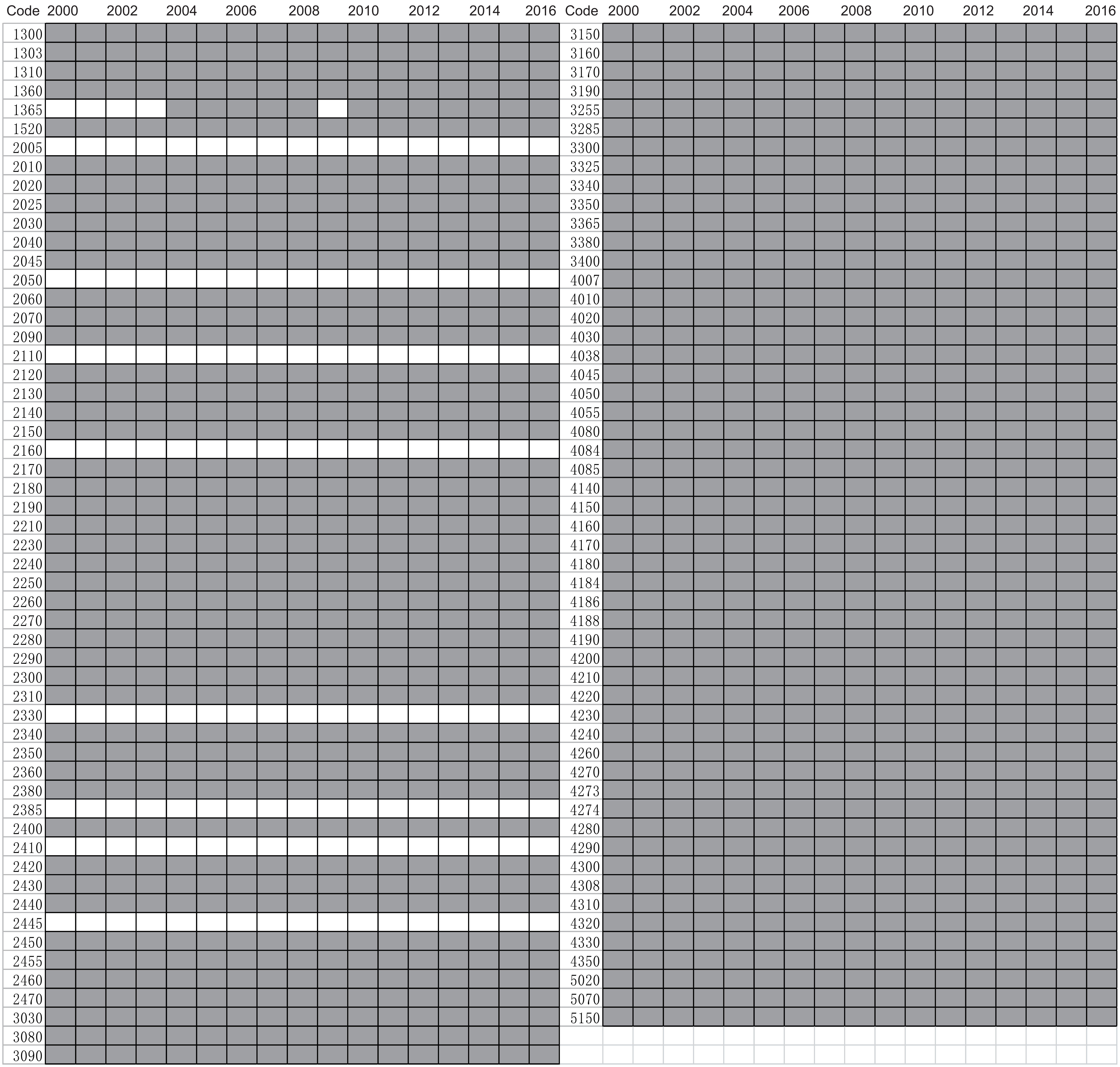

Data are sorted by country code, grey cells denote available data and white cells denote unavailable data

Appendix S6: Description of joinpoint regression model

Analysis of the trend of time series data include moving averages model, regression model and autoregressive integrated moving average model, etc. These classical approaches were performed commonly based on the regression models such as linear model, exponential model and logarithmic model. Typically, such models can demonstrate the overall trend of time series data whereas not be able to show period segmental time trends. The joinpoint regression model first introduced and fully established by Kim and colleagues solve this problem in 2000. Joinpoint regression involves fitting multiple lines to a single set of data. This technique is also known as piecewise or segmented regression and is useful in detecting abrupt changes and describing shifting time trends.

The joinpoint regression model for the observations, $(x_1, y_1), \ldots, (x_n , y_n)$, where $x_1 \leq \ldots \leq x_n$ without loss of generality, may be written as

$$E[y|x] = \beta_0 + \beta_1 x + \delta_1 (x-\tau_1)^+ \ldots + \delta_\kappa (x-\tau_\kappa)^+$$

where *y* is the outcome of interest, *x* is the calendar year, the τ_κ's are the unknown joinpoints and $\alpha^+ = \alpha$ for $\alpha > 0$ and 0 otherwise.

In this study, joinpoint analysis was used to identify years (as the independent variable) at which significant changes in mortality rate occurred over the study period and the size of these changes (annual percent change). The use of a natural log-linear model enables the analysis of a constant percentage change in rate over time. We allowed a maximum of 4 joinpoints for estimation as suggested by the program developers and used “Permutation test” option to select the best-fitted model. All statistical analyses were performed using the help of JoinPoint Regression Program version 4.1.0. A P-value smaller than 0.05 was considered a significant change in trend.

Appendix S7. Crude and age-standardized proportions and rates of diabetes microvaslular complication related deaths, by country

Country Code	Complication deaths	Total deaths	Midyear Pop (thousands)	Proportions				Rates			
				Crude	95%CI	Age-standardized	95%CI	Crude	95%CI	Age-Standardized	95%CI
1300	7796	25831	20 959	301.81	296.21-307.41	237.71	232.52-242.9	371.96	365.41-378.5	496.90	490.13-503.67
1303	21	132	3 350	159.09	96.69-221.49	121.12	65.46-176.78	6.27	3.6-8.94	19.08	14.45-23.71
1310	1493	30018	539 228	49.74	47.28-52.2	40.49	38.26-42.72	2.77	2.63-2.91	6.65	6.43-6.86
1360	511	3822	13 792	133.70	122.91-144.49	103.40	93.75-113.06	37.05	33.9-40.2	52.39	48.67-56.11
1365	134	511	466.674	262.23	224.09-300.37	210.25	174.92-245.59	287.14	246.09-328.19	409.48	364.87-454.1
1520	346	3638	177 618	95.11	85.57-104.64	82.35	73.42-91.28	1.95	1.74-2.15	3.83	3.54-4.12
2005	14	89	220.692	157.30	81.66-232.95	107.68	43.28-172.09	63.44	31.28-95.6	0.00	-
2010	71	705	1 451	100.71	78.49-122.92	84.64	64.09-105.18	48.92	37.82-60.02	78.24	64.42-92.06
2020	31582	145897	682 227	216.47	214.35-218.58	178.82	176.85-180.79	46.29	45.79-46.79	53.62	53.08-54.15
2025	106	598	1 702	177.26	146.65-207.87	149.16	120.6-177.71	62.29	50.8-73.77	77.85	65.12-90.58
2030	299	1466	5 795	203.96	183.33-224.58	163.45	144.52-182.38	51.60	45.9-57.29	95.34	87.78-102.9
2040	190	2995	4 750	63.44	54.71-72.17	48.67	40.96-56.37	40.00	34.43-45.58	32.41	27.37-37.45
2045	307	2092	5 225	146.75	131.59-161.91	116.92	103.15-130.69	58.75	52.37-65.13	149.22	139.56-158.88
2050	54	405	1045.644	133.33	100.23-166.44	120.88	89.13-152.62	51.64	38.23-65.06	0.00	-
2060	58	716	165 298	81.01	61.02-100.99	69.79	51.13-88.45	0.35	0.26-0.44	0.62	0.5-0.74
2070	220754	937312	3 254 631	235.52	234.66-236.38	192.04	191.24-192.84	67.83	67.55-68.1	113.33	112.99-113.68
2090	24997	124064	567 645	201.48	199.25-203.72	160.12	158.08-162.16	44.04	43.5-44.57	35.76	35.28-36.25
2110	14	74	969.505	189.19	99.95-278.43	154.79	72.38-237.21	14.44	6.93-21.95	0.00	-
2120	21954	60663	284 275	361.90	358.08-365.73	304.32	300.66-307.98	77.23	76.25-78.21	97.88	96.78-98.97
2130	35005	121777	749 032	287.45	284.91-289.99	236.44	234.06-238.83	46.73	46.26-47.21	81.22	80.6-81.83
2140	7073	12270	75 689	576.45	567.7-585.19	478.38	469.54-487.21	93.45	91.37-95.52	146.03	143.51-148.54
2150	15170	34034	191 177	445.73	440.45-451.01	364.96	359.85-370.08	79.35	78.14-80.56	76.71	75.52-77.9
2160	64	662	1200.76	96.68	74.16-119.19	94.55	72.26-116.83	53.30	40.59-66.01	0.00	-
2170	4557	17020	160 641	267.74	261.09-274.4	216.28	210.09-222.46	28.37	27.56-29.18	50.22	49.15-51.28
2180	21275	61030	247 289	348.60	344.82-352.38	283.32	279.75-286.9	86.03	84.93-87.14	159.79	158.35-161.23
2190	1271	20839	104 197	60.99	57.74-64.24	49.16	46.22-52.09	12.20	11.53-12.86	20.67	19.81-21.54
2210	67	391	3 721	171.36	134-208.71	130.69	97.28-164.1	18.01	13.73-22.28	51.07	44-58.15
2230	211	1343	1 801	157.11	137.65-176.57	127.33	109.5-145.15	117.13	102.28-131.97	162.95	145.89-180
2240	292	2312	6 912	126.30	112.76-139.84	92.59	80.78-104.41	42.24	37.5-46.99	35.65	31.28-40.02
2250	9692	54548	238 803	177.68	174.47-180.89	141.83	138.91-144.76	40.59	39.79-41.38	101.23	100.02-102.44
2260	606	5832	12 779	103.91	96.08-111.74	75.80	69.01-82.6	47.42	43.74-51.11	91.99	86.98-97.01
2270	53	614	164 040	86.32	64.11-108.53	65.66	46.07-85.26	0.32	0.24-0.41	0.94	0.79-1.09
2280	780	2472	135 255	315.53	297.21-333.85	254.45	237.28-271.62	5.77	5.36-6.17	14.63	13.99-15.27
2290	2131	23317	47 307	91.39	87.69-95.09	81.81	78.29-85.33	45.05	43.18-46.92	62.68	60.5-64.86
2300	292	2062	6 643	141.61	126.56-156.66	115.27	101.49-129.06	43.95	39.02-48.88	32.95	28.65-37.24
2310	483660	1253799	1 885 930	385.76	384.9-386.61	307.57	306.77-308.38	256.46	255.83-257.08	445.82	445.11-446.53
2340	9232	23170	96 489	398.45	392.14-404.75	311.84	305.88-317.81	95.68	93.82-97.54	221.20	218.58-223.82
2350	4853	15452	59 881	314.07	306.75-321.39	256.44	249.56-263.33	81.04	78.86-83.23	136.01	133.26-138.75
2360	7106	28126	103 208	252.65	247.57-257.73	205.50	200.77-210.22	68.85	67.31-70.4	142.38	140.25-144.51
2370	4098	41294	486 073	99.24	96.36-102.12	81.65	79.01-84.29	8.43	8.17-8.69	16.06	15.7-16.41
2380	6978	48099	60 674	145.08	141.93-148.22	120.94	118.03-123.86	115.01	112.47-117.55	102.94	100.52-105.35
2385	93	491	816.949	189.41	154.75-224.07	160.11	127.67-192.55	113.84	92.06-135.62	0.00	-
2400	192	1563	2 877	122.84	106.57-139.11	105.16	89.95-120.37	66.75	57.63-75.87	90.35	79.87-100.82
2410	1	8	105.257	125.00	-104.18-354.18	73.11	-107.28-253.5	9.50	-9.03-28.03	0.00	-
2420	135	1427	1 844	94.60	79.42-109.79	76.71	62.9-90.52	73.20	61.32-85.09	110.13	95.84-124.42
2430	761	2582	8 798	294.73	277.15-312.32	223.36	207.29-239.42	86.50	80.63-92.37	145.15	137.79-152.51
2440	1909	18182	22 406	104.99	100.54-109.45	81.34	77.37-85.32	85.20	81.55-88.86	115.86	111.67-120.05
2445	7	51	510.515	137.25	42.81-231.7	123.49	33.2-213.79	13.71	3.62-23.8	0.00	-
2450	255943	1253190	5 152 531	204.23	203.53-204.94	164.33	163.68-164.98	49.67	49.49-49.86	43.15	42.98-43.33
2455	155	572	1 816	270.98	234.55-307.4	215.99	182.27-249.71	85.37	72.52-98.23	90.40	77.21-103.59
2460	2041	11810	57 018	172.82	166-179.64	146.74	140.36-153.12	35.80	34.27-37.32	30.79	29.37-32.21
2470	49010	127233	468 130	385.20	382.52-387.87	309.73	307.19-312.27	104.69	103.82-105.57	201.07	199.92-202.22

Appendix S7 (continue). Crude and age-standardized proportions and rates of diabetes microvaslular complication related deaths, by country

Country Code	Complication deaths	Total deaths	Midyear Pop (thousands)	Proportions				Rates			
				Crude	95%CI	Age-standardized	95%CI	Crude	95%CI	Age-Standardized	95%CI
3030	67	149	6 437	449.66	369.79-529.54	357.73	280.77-434.7	10.41	7.93-12.89	26.61	22.68-30.54
3080	638	4521	18 201	141.12	130.97-151.27	132.05	122.18-141.92	35.05	32.38-37.73	37.71	34.95-40.48
3090	2485	8585	117 333	289.46	279.86-299.05	227.35	218.48-236.21	21.18	20.36-22	18.64	17.87-19.42
3150	5807	41113	119 244	141.24	137.88-144.61	114.08	111.01-117.15	48.70	47.48-49.92	53.10	51.82-54.37
3160	124221	234000	2 179 426	530.86	528.84-532.88	443.23	441.22-445.24	57.00	56.69-57.3	31.90	31.67-32.14
3170	1398	7196	117 407	194.27	185.13-203.42	156.75	148.35-165.15	11.91	11.29-12.53	36.26	35.19-37.32
3190	553	3497	47 791	158.14	146.04-170.23	128.45	117.36-139.54	11.57	10.61-12.53	152.41	149.18-155.63
3255	30	285	6 077	105.26	69.63-140.89	90.74	57.39-124.08	4.94	3.17-6.7	13.00	10.16-15.85
3285	56	791	51 435	70.80	52.92-88.67	46.68	31.98-61.38	1.09	0.8-1.37	4.15	3.6-4.71
3300	61149	160879	1 545 640	380.09	377.72-382.47	291.88	289.66-294.11	39.56	39.25-39.87	93.47	93.01-93.93
3325	4475	9862	837 138	453.76	443.94-463.59	360.51	351.03-369.99	5.35	5.19-5.5	6.93	6.75-7.11
3340	322	810	445 253	397.53	363.83-431.23	337.55	304.98-370.11	0.72	0.64-0.8	2.17	2.03-2.31
3350	1290	1346	81 495	958.40	947.73-969.06	759.62	736.79-782.45	15.83	14.97-16.69	23.83	22.78-24.88
3365	109	8535	339 127	12.77	10.39-15.15	10.86	8.66-13.06	0.32	0.26-0.38	0.45	0.38-0.52
3380	2880	45573	1 127 733	63.20	60.96-65.43	48.65	46.68-50.63	2.55	2.46-2.65	2.91	2.81-3.01
3400	15098	129437	1 205 594	116.64	114.89-118.39	99.42	97.79-101.05	12.52	12.32-12.72	21.66	21.4-21.92
4007	1178	2485	50 151	474.04	454.41-493.68	377.64	358.58-396.71	23.49	22.16-24.81	25.46	24.08-26.84
4010	19832	47617	142 224	416.49	412.06-420.92	312.87	308.7-317.03	139.44	137.64-141.24	91.91	90.41-93.41
4020	7536	27825	183 423	270.84	265.61-276.06	200.33	195.63-205.03	41.09	40.18-41.99	26.40	25.66-27.13
4030	660	4677	128 261	141.12	131.14-151.09	114.95	105.81-124.09	5.15	4.75-5.54	3.54	3.21-3.86
4038	7935	20877	73 721	380.08	373.5-386.67	296.38	290.18-302.57	107.64	105.4-109.87	76.37	74.46-78.29
4045	6031	37350	177 201	161.47	157.74-165.2	131.17	127.75-134.6	34.03	33.19-34.88	27.00	26.25-27.76
4050	3545	21386	93 673	165.76	160.78-170.75	134.15	129.59-138.72	37.84	36.62-39.07	27.17	26.13-28.21
4055	341	806	22 886	423.08	388.97-457.18	326.33	293.96-358.7	14.90	13.33-16.47	10.80	9.46-12.14
4080	20848	190007	1 055 276	109.72	108.32-111.13	76.07	74.88-77.26	19.76	19.49-20.02	12.32	12.11-12.53
4084	1323	9240	70 579	143.18	136.04-150.32	113.10	106.64-119.56	18.74	17.74-19.75	15.93	15-16.85
4085	117841	393755	1 383 230	299.27	297.84-300.71	225.43	224.12-226.73	85.19	84.73-85.66	51.55	51.18-51.92
4140	2791	5209	186 597	535.80	522.26-549.35	405.05	391.72-418.38	14.96	14.41-15.51	9.62	9.18-10.07
4150	8974	45633	169 811	196.66	193.01-200.3	159.51	156.15-162.87	52.85	51.78-53.91	39.63	38.7-40.55
4160	19	233	5 242	81.55	46.4-116.69	50.87	22.65-79.08	3.62	2-5.25	3.30	1.75-4.86
4170	1254	4548	73 523	275.73	262.74-288.71	232.49	220.22-244.77	17.06	16.12-17.99	18.00	17.04-18.96
4180	50937	286056	1 000 474	178.07	176.66-179.47	146.60	145.3-147.89	50.91	50.48-51.34	28.35	28.02-28.67
4184	2051	5799	91 072	353.68	341.38-365.99	275.31	263.82-286.81	22.52	21.56-23.48	41.86	40.56-43.16
4186	1113	3839	36 971	289.92	275.57-304.27	242.82	229.26-256.39	30.10	28.36-31.85	21.60	20.12-23.08
4188	1564	4796	54 557	326.11	312.84-339.37	263.49	251.03-275.96	28.67	27.27-30.07	21.50	20.28-22.72
4190	291	992	8 401	293.35	265.01-321.68	223.44	197.52-249.36	34.64	30.73-38.55	28.32	24.77-31.86
4200	753	1919	7 012	392.39	370.54-414.24	327.04	306.05-348.03	107.39	100.14-114.63	86.06	79.5-92.63
4210	10538	56174	280 920	187.60	184.37-190.82	138.62	135.77-141.48	37.51	36.81-38.22	28.52	27.91-29.14
4220	1802	11909	81 880	151.31	144.88-157.75	118.94	113.13-124.76	22.01	21-23.01	15.39	14.55-16.24
4230	26422	108121	651 318	244.37	241.81-246.94	194.61	192.25-196.97	40.57	40.09-41.05	35.35	34.9-35.79
4240	14331	54132	178 026	264.74	261.03-268.46	214.68	211.22-218.13	80.50	79.24-81.76	51.70	50.67-52.72
4260	2062	3599	70 081	572.94	556.78-589.1	438.68	422.47-454.89	29.42	28.17-30.67	31.02	29.74-32.3
4270	3590	37504	355 358	95.72	92.75-98.7	78.43	75.71-81.15	10.10	9.77-10.43	7.87	7.58-8.16
4273	422	3033	154 747	139.14	126.82-151.45	118.72	107.21-130.23	2.73	2.47-2.99	2.29	2.05-2.53
4274	583	3311	91 952	176.08	163.11-189.05	136.67	124.97-148.37	6.34	5.83-6.85	6.05	5.55-6.55
4280	29557	167833	764 891	176.11	174.29-177.93	149.01	147.31-150.72	38.64	38.21-39.07	24.60	24.25-24.94
4290	7782	33101	157 800	235.10	230.53-239.67	185.29	181.1-189.47	49.32	48.25-50.38	29.79	28.95-30.63
4300	5546	24210	130 569	229.08	223.79-234.37	165.83	161.14-170.52	42.48	41.38-43.57	27.61	26.72-28.5
4308	32055	103756	1 058 918	308.95	306.13-311.76	258.66	255.99-261.32	30.27	29.95-30.6	20.74	20.46-21.01
4310	26461	88766	932278.352	298.10	295.09-301.11	253.39	250.53-256.26	28.38	28.05-28.72	16.28	16.02-16.54
4320	744	3055	30107.108	243.54	228.31-258.76	195.89	181.82-209.97	24.71	22.96-26.47	25.42	23.64-27.2
4330	5073	12553	88522.74	404.13	395.54-412.71	307.03	298.96-315.1	57.31	55.78-58.84	41.91	40.59-43.23
4350	2094	8019	10 550	261.13	251.52-270.74	210.11	201.19-219.03	198.48	190.87-206.09	174.86	167.62-182.11
5020	15235	62964	364 439	241.96	238.62-245.31	193.29	190.21-196.38	41.80	41.15-42.45	35.10	34.5-35.7
5070	476	11880	14 320	40.07	36.54-43.59	28.91	25.9-31.92	33.24	30.3-36.18	137.17	131.53-142.8
5150	7205	13216	72 605	545.17	536.68-553.66	463.46	454.96-471.96	99.23	97.06-101.41	89.03	86.96-91.1

Note: data are sorted by country code; - denotes no available data

Appendix S8. Crude and age-standardized odd ratios of proportions and rates compared to overall, by country

Country Code	Proportions					Rates			
	Complication deaths	Crude OR	95% CI	Age-standardized OR	95% CI	Crude OR	95% CI	Age-standardized OR	95% CI
1300	7796	1.13	1.1-1.16	1.08	1.06-1.11	6.84	6.67-7.02	7.15	6.98-7.32
1303	21	0.59	0.37-0.94	0.83	0.56-1.25	0.12	0.08-0.18	0.27	0.21-0.35
1310	1493	0.19	0.18-0.2	0.21	0.2-0.22	0.05	0.05-0.05	0.10	0.09-0.1
1360	511	0.50	0.45-0.55	0.51	0.46-0.56	0.68	0.62-0.74	0.75	0.7-0.81
1365	134	0.98	0.81-1.18	1.00	0.82-1.21	5.28	4.36-6.4	5.89	4.98-6.97
1520	346	0.35	0.32-0.4	0.36	0.32-0.4	0.04	0.03-0.04	0.06	0.05-0.06
2005	14	0.59	0.33-1.03	0.59	0.33-1.04	1.17	0.68-2	0.00	-
2010	71	0.38	0.29-0.48	0.40	0.31-0.5	0.90	0.71-1.14	1.13	0.93-1.36
2020	31582	0.81	0.8-0.82	0.82	0.81-0.83	0.85	0.84-0.86	0.77	0.76-0.78
2025	106	0.66	0.54-0.81	0.68	0.55-0.84	1.15	0.94-1.39	1.12	0.94-1.34
2030	299	0.76	0.67-0.86	0.80	0.7-0.9	0.95	0.84-1.07	1.37	1.26-1.5
2040	190	0.24	0.2-0.27	0.24	0.21-0.28	0.74	0.64-0.85	0.47	0.4-0.55
2045	307	0.55	0.49-0.62	0.52	0.46-0.59	1.08	0.96-1.21	2.15	1.99-2.31
2050	54	0.50	0.37-0.66	0.53	0.4-0.7	0.95	0.72-1.25	0.00	-
2060	58	0.30	0.23-0.39	0.27	0.2-0.36	0.01	0-0.01	0.01	0.01-0.01
2070	220754	0.88	0.87-0.88	0.89	0.88-0.89	1.25	1.24-1.25	1.63	1.62-1.64
2090	24997	0.75	0.74-0.76	0.79	0.77-0.8	0.81	0.8-0.82	0.51	0.51-0.52
2110	14	0.71	0.4-1.25	0.79	0.45-1.37	0.27	0.16-0.45	0.00	-
2120	21954	1.35	1.33-1.37	1.39	1.37-1.41	1.42	1.4-1.44	1.41	1.39-1.43
2130	35005	1.07	1.06-1.09	1.10	1.08-1.11	0.86	0.85-0.87	1.17	1.16-1.18
2140	7073	2.15	2.09-2.21	2.19	2.13-2.26	1.72	1.68-1.76	2.10	2.06-2.14
2150	15170	1.66	1.63-1.7	1.67	1.64-1.71	1.46	1.44-1.48	1.10	1.08-1.12
2160	64	0.36	0.28-0.47	0.42	0.33-0.53	0.98	0.76-1.26	0.00	-
2170	4557	1.00	0.97-1.03	0.96	0.93-0.99	0.52	0.51-0.54	0.72	0.71-0.74
2180	21275	1.30	1.28-1.32	1.31	1.29-1.33	1.58	1.56-1.6	2.30	2.27-2.32
2190	1271	0.23	0.22-0.24	0.23	0.21-0.24	0.22	0.21-0.24	0.30	0.28-0.31
2210	67	0.64	0.49-0.83	0.66	0.51-0.86	0.33	0.26-0.42	0.73	0.63-0.85
2230	211	0.59	0.51-0.68	0.61	0.53-0.71	2.15	1.87-2.48	2.34	2.07-2.65
2240	292	0.47	0.42-0.53	0.47	0.41-0.53	0.78	0.69-0.87	0.51	0.45-0.58
2250	9692	0.66	0.65-0.68	0.64	0.63-0.66	0.75	0.73-0.76	1.46	1.44-1.48
2260	606	0.39	0.36-0.42	0.37	0.34-0.4	0.87	0.8-0.95	1.32	1.25-1.4
2270	53	0.32	0.24-0.43	0.34	0.26-0.45	0.01	0-0.01	0.01	0.01-0.02
2280	780	1.18	1.09-1.28	1.25	1.16-1.36	0.11	0.1-0.11	0.21	0.2-0.22
2290	2131	0.34	0.33-0.36	0.37	0.35-0.38	0.83	0.79-0.87	0.90	0.87-0.94
2300	292	0.53	0.47-0.6	0.55	0.49-0.62	0.81	0.72-0.91	0.47	0.41-0.54
2310	483660	1.44	1.43-1.44	1.38	1.37-1.38	4.72	4.7-4.73	6.41	6.39-6.43
2330	7205	1.49	1.45-1.52	1.90	1.39-1.46	1.76	1.72-1.8	3.18	3.13-3.23
2340	1802	1.17	1.13-1.21	1.42	1.17-1.24	1.49	1.45-1.53	1.96	1.91-2
2350	7106	0.94	0.92-0.97	1.20	0.89-0.94	1.27	1.24-1.3	2.05	2.01-2.08
2360	4098	0.37	0.36-0.38	0.91	0.36-0.39	0.16	0.15-0.16	0.23	0.23-0.24
2380	6978	0.54	0.53-0.56	0.56	0.55-0.58	2.12	2.06-2.17	1.48	1.44-1.52
2385	93	0.71	0.57-0.88	0.75	0.6-0.93	2.09	1.69-2.59	0.00	-
2400	192	0.46	0.39-0.53	0.48	0.42-0.56	1.23	1.06-1.42	1.30	1.14-1.48
2410	1	0.47	0.06-3.73	0.28	0.02-4.01	0.17	0.02-1.25	0.00	-
2420	135	0.35	0.3-0.42	0.37	0.31-0.44	1.35	1.13-1.6	1.58	1.37-1.83
2430	761	1.10	1.01-1.19	1.05	0.97-1.15	1.59	1.48-1.71	2.09	1.97-2.21
2440	1909	0.39	0.37-0.41	0.39	0.38-0.41	1.57	1.5-1.64	1.67	1.6-1.74
2445	7	0.51	0.23-1.13	0.48	0.21-1.09	0.25	0.12-0.53	0.00	-
2450	255943	0.76	0.76-0.77	0.79	0.79-0.79	0.91	0.91-0.92	0.62	0.62-0.62
2455	155	1.01	0.85-1.21	1.02	0.85-1.22	1.57	1.33-1.85	1.30	1.11-1.53
2460	2041	0.64	0.62-0.68	0.68	0.65-0.71	0.66	0.63-0.69	0.44	0.42-0.46

Data were sorted by country code; - denotes no available data

Appendix S8 (continue). Crude and age-standardized odd ratios of proportions and rates compared to overall, by country									
Country Code	Complication deaths	Proportions				Rates			
		Crude OR	95% CI	Age-standardized OR	95% CI	Crude OR	95% CI	Age-standardized OR	95% CI
2470	49010	1.44	1.42-1.45	1.41	1.4-1.43	1.93	1.91-1.94	2.89	2.87-2.91
3030	67	1.68	1.26-2.24	1.53	1.14-2.07	0.19	0.15-0.24	0.38	0.33-0.45
3080	638	0.53	0.48-0.57	0.61	0.57-0.66	0.64	0.6-0.7	0.54	0.5-0.59
3090	2485	1.08	1.03-1.13	1.02	0.97-1.06	0.39	0.37-0.41	0.27	0.26-0.28
3150	5807	0.53	0.51-0.54	0.50	0.49-0.52	0.90	0.87-0.92	0.76	0.74-0.78
3160	124221	1.98	1.97-1.99	2.05	2.04-2.07	1.05	1.04-1.05	0.46	0.46-0.46
3170	1398	0.72	0.68-0.77	0.68	0.64-0.72	0.22	0.21-0.23	0.52	0.51-0.54
3190	553	0.59	0.54-0.65	0.61	0.56-0.67	0.21	0.2-0.23	2.19	2.14-2.25
3255	30	0.39	0.27-0.57	0.37	0.25-0.55	0.09	0.06-0.13	0.19	0.15-0.23
3285	4853	0.26	0.2-0.35	0.33	0.26-0.42	0.02	0.02-0.03	0.06	0.05-0.07
3300	61149	1.42	1.4-1.43	1.34	1.33-1.35	0.73	0.72-0.73	1.34	1.34-1.35
3325	4475	1.69	1.63-1.75	1.71	1.65-1.78	0.10	0.1-0.1	0.10	0.1-0.1
3340	322	1.48	1.3-1.69	1.64	1.44-1.86	0.01	0.01-0.01	0.03	0.03-0.03
3350	1290	3.58	3.31-3.86	3.37	3.12-3.65	0.29	0.28-0.31	0.34	0.33-0.36
3365	109	0.05	0.04-0.06	0.05	0.04-0.06	0.01	0-0.01	0.01	0.01-0.01
3380	2880	0.24	0.23-0.24	0.22	0.21-0.23	0.05	0.05-0.05	0.04	0.04-0.04
3400	15098	0.44	0.43-0.44	0.45	0.44-0.46	0.23	0.23-0.23	0.31	0.31-0.32
4007	1178	1.77	1.65-1.9	1.73	1.61-1.85	0.43	0.41-0.46	0.37	0.35-0.39
4010	19832	1.55	1.53-1.58	1.53	1.5-1.56	2.56	2.53-2.6	1.32	1.3-1.35
4020	7536	1.01	0.99-1.04	0.99	0.96-1.01	0.76	0.74-0.77	0.38	0.37-0.39
4030	660	0.53	0.49-0.57	0.53	0.48-0.57	0.09	0.09-0.1	0.05	0.05-0.06
4038	7935	1.42	1.38-1.46	1.44	1.4-1.47	1.98	1.93-2.03	1.10	1.07-1.13
4045	6031	0.60	0.59-0.62	0.61	0.6-0.63	0.63	0.61-0.64	0.39	0.38-0.4
4050	3545	0.62	0.6-0.64	0.63	0.61-0.66	0.70	0.67-0.72	0.39	0.38-0.41
4055	341	1.58	1.39-1.79	1.62	1.43-1.84	0.27	0.25-0.3	0.16	0.14-0.18
4080	20848	0.41	0.4-0.42	0.39	0.38-0.4	0.36	0.36-0.37	0.18	0.17-0.18
4084	1323	0.53	0.5-0.57	0.52	0.49-0.55	0.34	0.33-0.36	0.23	0.22-0.24
4085	117841	1.12	1.11-1.12	1.10	1.09-1.11	1.57	1.56-1.58	0.74	0.74-0.75
4140	2791	2.00	1.91-2.09	2.03	1.94-2.12	0.28	0.27-0.29	0.14	0.13-0.14
4150	8974	0.73	0.72-0.75	0.75	0.73-0.77	0.97	0.95-0.99	0.57	0.56-0.58
4160	19	0.30	0.19-0.49	0.27	0.16-0.44	0.07	0.04-0.1	0.05	0.03-0.08
4170	1254	1.03	0.97-1.1	1.09	1.02-1.16	0.31	0.3-0.33	0.26	0.25-0.27
4180	50937	0.66	0.66-0.67	0.70	0.69-0.7	0.94	0.93-0.94	0.41	0.4-0.41
4184	2051	1.32	1.25-1.39	1.18	1.12-1.25	0.41	0.4-0.43	0.60	0.58-0.62
4186	1113	1.08	1.01-1.16	1.11	1.04-1.19	0.55	0.52-0.59	0.31	0.29-0.33
4188	1564	1.22	1.15-1.29	1.26	1.19-1.34	0.53	0.5-0.55	0.31	0.29-0.33
4190	291	1.09	0.96-1.25	1.08	0.95-1.24	0.64	0.57-0.72	0.41	0.36-0.46
4200	753	1.46	1.35-1.59	1.54	1.42-1.68	1.98	1.83-2.13	1.24	1.14-1.34
4210	10538	0.70	0.69-0.71	0.69	0.67-0.7	0.69	0.68-0.7	0.41	0.4-0.42
4220	56	0.56	0.54-0.59	0.58	0.55-0.61	0.40	0.39-0.42	0.22	0.21-0.23
4230	26422	0.91	0.9-0.92	0.93	0.92-0.94	0.75	0.74-0.76	0.51	0.5-0.52
4240	14331	0.99	0.97-1.01	1.04	1.02-1.05	1.48	1.46-1.51	0.74	0.73-0.76
4260	2062	2.14	2.03-2.26	2.14	2.03-2.26	0.54	0.52-0.57	0.45	0.43-0.47
4270	3590	0.36	0.35-0.37	0.35	0.34-0.36	0.19	0.18-0.19	0.11	0.11-0.12
4273	422	0.52	0.47-0.57	0.54	0.49-0.6	0.05	0.05-0.06	0.03	0.03-0.04
4274	583	0.66	0.6-0.72	0.66	0.6-0.72	0.12	0.11-0.13	0.09	0.08-0.09
4280	29557	0.66	0.65-0.67	0.70	0.7-0.71	0.71	0.7-0.72	0.35	0.35-0.36
4290	7782	0.88	0.86-0.9	0.89	0.87-0.91	0.91	0.89-0.93	0.43	0.42-0.44
4300	5546	0.85	0.83-0.88	0.82	0.8-0.85	0.78	0.76-0.8	0.40	0.38-0.41
4308	32055	1.15	1.14-1.17	1.21	1.2-1.23	0.56	0.55-0.56	0.30	0.29-0.3
4310	26461	1.11	1.1-1.13	1.18	1.17-1.2	0.52	0.52-0.53	0.23	0.23-0.24
4320	744	0.91	0.84-0.98	0.93	0.86-1.01	0.45	0.42-0.49	0.37	0.34-0.39
4330	5073	1.51	1.46-1.56	1.53	1.48-1.58	1.05	1.02-1.08	0.60	0.58-0.62
4350	2094	0.97	0.93-1.02	0.94	0.9-0.99	3.65	3.48-3.83	2.51	2.39-2.64
5020	15235	0.90	0.89-0.92	0.93	0.91-0.95	0.77	0.76-0.78	0.50	0.5-0.51
5070	476	0.15	0.14-0.16	0.14	0.12-0.15	0.61	0.56-0.67	1.97	1.88-2.07
5150	7205	2.03	1.98-2.09	2.12	2.06-2.18	1.83	1.78-1.87	1.28	1.25-1.31

Data were sorted by country code; - denotes no available data

Appendix S9. Crude and age-standardized proportions from 2000 to 2016, by sex

Year	Total				Male				Female			
	Crude	95% CI	Age-standardized	95% CI	Crude	95% CI	Age-standardized	95% CI	Crude	95% CI	Age-standardized	95% CI
**2000**	225.11	223.71-226.52	229.15	227.73-230.56	242.05	239.88-244.21	239.82	237.66-241.98	218.92	217.07-220.78	213.44	211.6-215.28
**2001**	232.97	231.59-234.35	235.63	234.24-237.02	246.08	243.97-248.19	244.40	242.29-246.50	227.11	225.27-228.95	223.60	221.77-225.42
**2002**	228.87	227.55-230.18	231.55	230.23-232.88	243.30	241.28-245.32	241.70	239.69-243.72	222.02	220.27-223.78	218.39	216.65-220.14
**2003**	231.19	229.85-232.52	232.11	230.78-233.45	241.93	239.91-243.95	240.97	238.95-242.99	223.95	222.18-225.73	222.97	221.20-224.75
**2004**	226.18	224.83-227.53	226.75	225.40-228.10	235.51	233.48-237.55	234.85	232.82-236.88	219.41	217.61-221.22	218.81	217.00-220.61
**2005**	226.22	224.89-227.55	226.40	225.07-227.73	235.05	233.05-237.05	234.73	232.73-236.73	219.18	217.4-220.95	219.09	217.31-220.87
**2006**	237.56	236.26-238.86	238.85	237.55-240.15	248.44	246.5-250.38	247.60	245.66-249.54	230.54	228.78-232.29	228.91	227.16-230.66
**2007**	260.20	258.85-261.54	260.60	259.25-261.94	269.97	267.97-271.96	269.47	267.48-271.47	252.44	250.62-254.25	252.32	250.50-254.13
**2008**	264.42	263.06-265.79	263.41	262.05-264.78	273.32	271.29-275.35	273.80	271.77-275.83	254.81	252.96-256.66	256.39	254.54-258.24
**2009**	256.89	255.60-258.18	257.20	255.92-258.49	269.07	267.16-270.98	268.63	266.72-270.54	246.79	245.05-248.52	246.76	245.02-248.50
**2010**	263.30	262.02-264.58	263.01	261.73-264.29	274.86	272.96-276.76	274.94	273.04-276.85	252.58	250.85-254.32	253.15	251.42-254.89
**2011**	289.80	288.49-291.11	289.45	288.14-290.76	301.33	299.4-303.26	301.44	299.51-303.37	278.84	277.06-280.61	279.48	277.70-281.26
**2012**	294.87	293.54-296.21	293.51	292.18-294.84	303.62	301.67-305.58	304.09	302.13-306.05	284.44	282.62-286.26	286.55	284.73-288.37
**2013**	314.78	313.43-316.13	313.42	312.07-314.77	325.74	323.76-327.71	326.16	324.18-328.14	302.21	300.37-304.06	304.22	302.38-306.07
**2014**	312.51	311.17-313.84	311.69	310.35-313.03	323.52	321.57-325.47	323.31	321.36-325.26	300.79	298.96-302.62	302.32	300.49-304.16
**2015**	318.27	316.93-319.60	316.70	315.36-318.03	328.34	326.40-330.28	328.38	326.43-330.32	305.87	304.03-307.71	308.57	306.73-310.41
**2016**	317.36	316.03-318.68	315.95	314.62-317.27	325.11	323.20-327.02	325.32	323.41-327.23	307.26	305.43-309.10	309.77	307.94-311.61
**Overall**	267.82	267.49-268.14	267.98	267.65-268.30	279.58	279.10-280.07	279.21	278.73-279.70	257.85	257.41-258.29	257.85	257.41-258.29

Data were sorted by year and sex; number were proportions with 95% CI (per 1000 diabetes deaths)

Appendix S10. Crude age specific proportions from 2000 to 2016, by sex												
	Total (years)				Male (years)				Female (years)			
**Year**	**0-19**	**20-44**	**45-64**	**>65**	**0-19**	**20-44**	**45-64**	**>65**	**0-19**	**20-44**	**45-64**	**>65**
**2000**	65.86	198.35	274.94	215.60	58.69	179.70	276.42	232.60	71.30	224.39	273.23	204.04
**2001**	77.49	218.00	283.59	221.37	66.01	201.88	277.50	236.63	82.12	240.41	290.50	210.76
**2002**	71.35	203.02	276.46	218.67	79.60	181.95	274.73	234.83	64.76	232.21	278.32	207.43
**2003**	66.22	211.30	279.59	218.94	67.45	190.25	273.57	233.04	64.84	241.66	286.57	208.87
**2004**	77.03	198.10	262.99	217.41	57.75	180.43	254.57	231.99	92.96	223.56	272.94	206.84
**2005**	70.03	193.02	260.46	217.92	69.08	176.11	254.59	231.40	70.56	217.71	267.66	208.21
**2006**	86.45	212.50	283.20	225.89	84.51	195.12	278.79	239.55	88.10	237.52	288.53	215.68
**2007**	84.47	240.51	319.64	243.09	89.47	220.61	308.22	257.51	79.63	269.27	334.31	232.35
**2008**	69.21	242.21	317.09	248.57	69.70	226.98	303.00	264.89	68.78	265.11	335.11	236.29
**2009**	92.66	248.95	311.34	240.47	112.24	238.80	301.69	257.52	76.77	262.71	323.72	227.61
**2010**	113.77	255.27	320.39	245.34	149.48	242.22	309.66	262.58	82.59	273.85	334.29	232.27
**2011**	84.72	274.94	337.52	275.11	97.14	263.48	328.38	292.73	74.59	291.27	349.45	261.46
**2012**	89.12	272.32	334.99	282.26	104.53	251.96	322.00	299.61	77.33	300.59	352.06	268.67
**2013**	89.71	284.93	351.95	303.52	93.33	266.94	341.78	323.21	86.61	310.38	365.26	287.77
**2014**	60.56	277.27	336.48	306.05	53.51	262.36	328.60	325.33	66.67	298.12	346.86	290.43
**2015**	59.86	280.27	350.18	308.82	47.79	260.27	338.23	328.55	70.95	308.05	366.24	292.59
**2016**	55.73	280.96	351.25	307.25	68.73	267.34	339.32	323.12	44.51	299.41	367.67	294.01
**Overall**	77.97	241.11	311.21	256.13	81.47	224.25	303.36	273.85	74.70	264.95	321.06	242.71

Data were sorted by year and age group; number were proportions (per 1000 diabetes deaths)

Appendix S11. Crude and age-standardized proportions from 2000 to 2016, by region

Year	Crude proportions								Age-standardized proportions							
	Africa	Asia	Caribbean	Europe	Middle East	North America	Oceania	South America	Africa	Asia	Caribbean	Europe	Middle East	North America	Oceania	South America
**2000**	23.64	340.56	86.17	199.10	62.36	217.62	304.54	242.24	23.79	346.50	84.44	195.64	65.31	219.63	301.72	237.66
**2001**	32.26	424.17	159.33	216.01	32.49	220.67	277.48	237.19	30.44	421.75	154.20	214.16	38.09	222.57	273.53	232.96
**2002**	43.25	352.23	166.78	216.33	129.11	210.31	273.04	241.84	43.88	356.39	161.30	213.90	133.17	211.79	270.24	238.17
**2003**	39.50	369.63	181.29	209.50	56.63	215.60	244.91	250.56	41.46	371.07	177.09	207.49	57.61	216.54	246.52	247.57
**2004**	37.14	350.23	182.06	212.83	89.65	217.67	230.73	244.65	36.20	350.54	179.61	211.41	98.10	218.56	232.38	242.19
**2005**	247.94	358.11	180.19	211.56	123.22	208.43	291.69	253.39	243.07	356.34	179.57	210.70	126.26	209.28	349.86	251.84
**2006**	260.87	304.29	172.08	207.79	117.45	235.69	241.71	254.51	253.72	308.28	170.36	207.31	124.90	235.67	240.86	253.76
**2007**	115.39	421.76	217.51	208.92	84.20	274.12	230.23	250.36	114.85	420.99	214.42	208.54	92.12	274.36	230.84	250.12
**2008**	133.74	513.16	221.14	218.35	110.05	285.94	186.23	256.70	132.63	510.83	218.87	217.95	117.24	285.54	187.23	256.62
**2009**	131.97	435.88	208.99	213.98	82.91	277.24	183.17	257.04	133.72	435.23	208.79	213.79	81.36	276.47	183.07	257.12
**2010**	154.23	456.34	229.09	216.12	79.80	282.62	204.31	257.65	152.89	455.57	230.93	216.39	80.04	282.37	206.33	258.84
**2011**	181.82	450.89	246.17	241.64	79.07	335.04	177.49	260.01	181.13	450.32	247.50	241.83	79.21	335.19	179.72	260.98
**2012**	183.30	505.92	253.30	250.11	159.16	341.52	165.72	267.16	184.99	504.63	255.74	250.21	157.88	341.22	166.61	268.66
**2013**	168.73	493.10	283.07	263.50	183.88	370.77	365.79	274.40	174.18	489.57	283.78	263.41	183.59	370.70	366.93	276.04
**2014**	224.25	502.85	291.71	288.58	177.15	345.15	389.69	273.54	223.64	498.65	297.87	288.89	177.45	344.30	392.01	275.76
**2015**	319.47	507.65	350.33	277.42	164.80	365.95	398.19	260.87	320.39	506.81	354.96	277.61	164.78	365.08	402.92	263.53
**2016**	344.91	497.02	346.81	256.00	149.07	365.60	371.27	262.24	337.97	491.15	350.61	255.68	150.02	364.45	373.83	264.80
**Overall**	161.10	413.10	222.70	231.90	127.40	287.30	260.00	255.90	160.90	412.80	222.40	231.30	128.00	287.50	262.20	255.40

Data were sorted by year and regions; number were proportions with 95% CI (per 1000 diabetes deaths)

Appendix S12. Crude and age-standardized rates from 2000 to 2016, by sex												
	**Total**				**Males**				**Females**			
**Year**	**Crude**	**95% CI**	**Age-standardized**	**95% CI**	**Crude**	**95% CI**	**Age-standardized**	**95% CI**	**Crude**	**95% CI**	**Age-standardized**	**95% CI**
**2000**	40.62	40.34-40.90	46.01	45.71-46.31	38.18	37.79-38.56	44.07	43.66-44.49	41.80	41.41-42.19	46.84	46.42-47.25
**2001**	43.69	43.40-43.98	48.81	48.51-49.12	40.97	40.58-41.37	46.47	46.05-46.90	45.05	44.64-45.45	49.90	49.48-50.33
**2002**	46.18	45.89-46.48	50.85	50.54-51.16	43.63	43.22-44.03	48.68	48.25-49.11	47.32	46.91-47.73	51.71	51.28-52.14
**2003**	45.22	44.93-45.51	49.21	48.91-49.51	42.77	42.37-43.17	47.06	46.64-47.48	46.27	45.87-46.68	50.06	49.64-50.49
**2004**	42.04	41.76-42.32	45.19	44.90-45.48	39.92	39.53-40.30	43.29	42.89-43.69	42.89	42.50-43.29	45.90	45.50-46.31
**2005**	42.91	42.63-43.19	45.62	45.34-45.91	40.79	40.40-41.18	43.68	43.28-44.08	43.75	43.36-44.15	46.35	45.95-46.76
**2006**	48.53	48.23-48.82	50.56	50.26-50.86	47.07	46.65-47.48	49.25	48.83-49.68	48.59	48.18-49.00	50.52	50.10-50.94
**2007**	52.32	52.02-52.63	53.60	53.29-53.91	50.68	50.25-51.10	52.02	51.59-52.45	52.48	52.05-52.90	53.70	53.27-54.13
**2008**	51.01	50.71-51.31	51.42	51.12-51.72	49.51	49.09-49.93	49.92	49.5-50.35	51.08	50.66-51.49	51.48	51.06-51.90
**2009**	54.90	54.59-55.21	54.50	54.19-54.81	53.91	53.48-54.35	53.44	53.01-53.88	54.39	53.96-54.82	54.02	53.59-54.44
**2010**	56.84	56.52-57.15	55.60	55.29-55.91	55.95	55.51-56.39	54.58	54.15-55.02	56.18	55.75-56.62	55.03	54.60-55.46
**2011**	63.18	62.85-63.51	60.65	60.33-60.98	62.42	61.96-62.88	59.62	59.16-60.07	62.23	61.77-62.68	59.89	59.45-60.34
**2012**	61.80	61.47-62.12	58.27	57.96-58.59	60.78	60.32-61.23	56.86	56.42-57.30	61.14	60.69-61.58	57.88	57.44-58.31
**2013**	66.25	65.92-66.58	61.39	61.07-61.71	65.83	65.36-66.30	60.40	59.95-60.85	64.88	64.42-65.33	60.42	59.97-60.86
**2014**	66.29	65.96-66.62	60.38	60.06-60.69	66.25	65.78-66.72	59.65	59.20-60.10	64.55	64.09-65.00	59.14	58.07-59.57
**2015**	67.68	67.34-68.01	60.69	60.37-61.01	67.94	67.46-68.41	60.14	59.69-60.58	65.61	65.15-66.06	59.24	58.80-59.67
**2016**	68.17	67.83-68.50	60.02	59.71-60.33	68.54	68.07-69.02	59.46	59.02-59.90	65.97	65.52-66.43	58.54	58.11-58.97
**Overall**	53.61	53.54-53.69	53.61	53.53-53.68	53.10	52.99-53.20	52.51	52.41-52.62	54.11	54.00-54.21	54.11	54.00-54.21

Data were sorted by year and sex; numbers denote rates with 95%CI (per 100 000 person years)

Appendix S13. Crude age specific rates from 2000 to 2016, by sex												
	Total (years)				Male (years)				Female (years)			
**Year**	**0-19**	**20-44**	**45-64**	**>65**	**0-19**	**20-44**	**45-64**	**>65**	**0-19**	**20-44**	**45-64**	**>65**
**2000**	0.10	3.93	61.24	257.32	0.08	4.12	65.52	268.96	0.13	3.70	56.79	248.15
**2001**	0.12	4.29	62.52	277.89	0.08	4.62	66.83	288.74	0.15	3.92	58.03	269.09
**2002**	0.11	4.18	65.23	291.64	0.10	4.33	70.73	304.08	0.11	3.99	59.56	281.57
**2003**	0.08	3.79	60.10	288.84	0.07	4.00	65.40	301.39	0.08	3.54	54.64	278.58
**2004**	0.09	3.34	52.77	270.57	0.06	3.56	56.76	285.62	0.12	3.08	48.60	258.53
**2005**	0.08	3.31	52.71	274.34	0.07	3.57	57.90	286.76	0.09	3.00	47.39	264.17
**2006**	0.11	4.15	64.17	295.09	0.09	4.47	70.97	313.49	0.12	3.77	57.21	280.35
**2007**	0.11	4.49	69.05	313.12	0.11	4.84	76.31	330.75	0.11	4.08	61.62	298.82
**2008**	0.08	4.07	62.42	310.44	0.07	4.54	68.11	331.39	0.09	3.55	56.53	293.62
**2009**	0.13	4.95	70.22	320.39	0.14	5.48	77.94	342.80	0.13	4.35	62.32	302.36
**2010**	0.15	4.96	71.70	327.34	0.18	5.48	79.52	351.04	0.12	4.37	63.65	308.25
**2011**	0.11	5.27	75.89	364.56	0.11	5.88	84.94	391.48	0.11	4.58	66.61	342.72
**2012**	0.10	4.80	68.88	359.93	0.09	5.13	76.46	386.82	0.10	4.41	61.06	338.00
**2013**	0.10	5.06	71.58	381.69	0.09	5.49	80.06	414.43	0.11	4.55	62.83	355.01
**2014**	0.06	4.99	69.23	377.96	0.05	5.43	78.20	411.91	0.08	4.47	60.01	350.24
**2015**	0.06	4.86	69.53	380.05	0.04	5.17	78.11	417.70	0.07	4.47	60.65	349.36
**2016**	0.06	5.18	71.83	370.66	0.06	5.58	81.51	404.75	0.05	4.69	61.85	342.60
**Overall**	0.10	4.46	66.27	325.81	0.09	4.82	73.28	349.26	0.10	4.04	59.06	306.99

Data were sorted by year and age group; numbers denote rates with 95%CI (per 100 000 person years)

Appendix S14. Crude and age-standardized rates from 2000 to 2016, by region

Year	Crude rates								Age-standardized rates							
	Africa	Asia	Caribbean	Europe	Middle East	North America	Oceania	South America	Africa	Asia	Caribbean	Europe	Middle East	North America	Oceania	South America
**2000**	1.08	29.70	12.80	23.69	1.63	61.63	49.62	76.24	1.33	36.38	14.63	27.04	1.89	68.71	56.21	91.86
**2001**	1.73	30.38	33.33	31.26	0.88	64.65	47.08	74.02	2.00	36.35	37.18	35.15	1.02	71.18	52.23	87.31
**2002**	2.22	34.21	33.24	34.92	3.29	64.52	56.12	76.78	2.59	39.75	36.63	38.49	3.80	70.32	61.33	88.87
**2003**	1.72	35.75	39.11	41.29	1.63	68.14	54.12	53.39	1.97	40.58	42.61	44.96	1.85	73.53	58.48	60.56
**2004**	2.31	21.43	43.28	38.60	2.58	68.97	51.70	53.08	2.53	24.20	46.60	41.54	2.87	73.63	54.79	59.06
**2005**	11.94	21.98	43.53	39.82	3.31	69.75	23.59	55.03	13.04	24.28	46.26	42.59	3.68	73.64	24.56	60.09
**2006**	13.87	42.17	40.22	37.59	3.06	77.16	51.06	57.57	14.77	44.49	42.01	39.24	3.26	80.38	52.58	61.36
**2007**	12.80	42.71	39.74	40.03	2.21	88.78	52.22	59.28	13.26	43.99	40.80	41.01	2.29	91.21	53.18	61.67
**2008**	14.31	21.31	40.83	42.16	3.76	95.47	44.79	61.44	14.50	21.49	41.29	42.41	3.81	96.73	45.05	62.46
**2009**	20.28	43.85	49.45	41.38	13.94	90.81	42.11	62.71	19.96	43.11	49.22	41.00	13.93	90.66	41.91	62.32
**2010**	16.55	45.00	59.25	41.47	13.48	95.51	35.28	66.01	16.00	43.22	58.07	40.61	13.19	94.01	34.70	64.15
**2011**	17.88	45.62	64.28	46.11	13.25	114.04	41.66	67.68	16.98	42.88	61.70	43.99	12.68	110.69	40.42	64.02
**2012**	20.88	22.03	63.14	49.02	25.59	118.22	38.25	69.93	19.32	19.80	59.50	45.71	24.21	113.01	36.45	64.50
**2013**	25.44	20.41	71.60	52.00	27.08	129.10	65.71	73.29	22.98	17.86	66.23	47.49	25.13	121.64	61.64	65.88
**2014**	25.54	20.55	65.07	56.40	25.01	124.18	69.24	74.39	22.74	17.49	59.05	50.56	22.78	115.21	63.94	65.23
**2015**	17.51	20.14	50.50	59.47	24.68	135.84	74.99	64.09	15.35	16.68	44.94	52.65	22.09	124.19	68.50	54.80
**2016**	18.71	33.79	50.83	50.40	21.26	138.52	59.68	65.25	15.91	27.45	44.29	43.31	18.61	124.75	53.36	54.30
**Overall**	13.60	31.20	42.80	72.60	11.90	95.80	50.80	65.30	13.00	31.60	46.80	42.30	11.20	94.70	50.60	66.00

Data were sorted by year and region; numbers denote rates with 95%CI (per 100 000 person years)

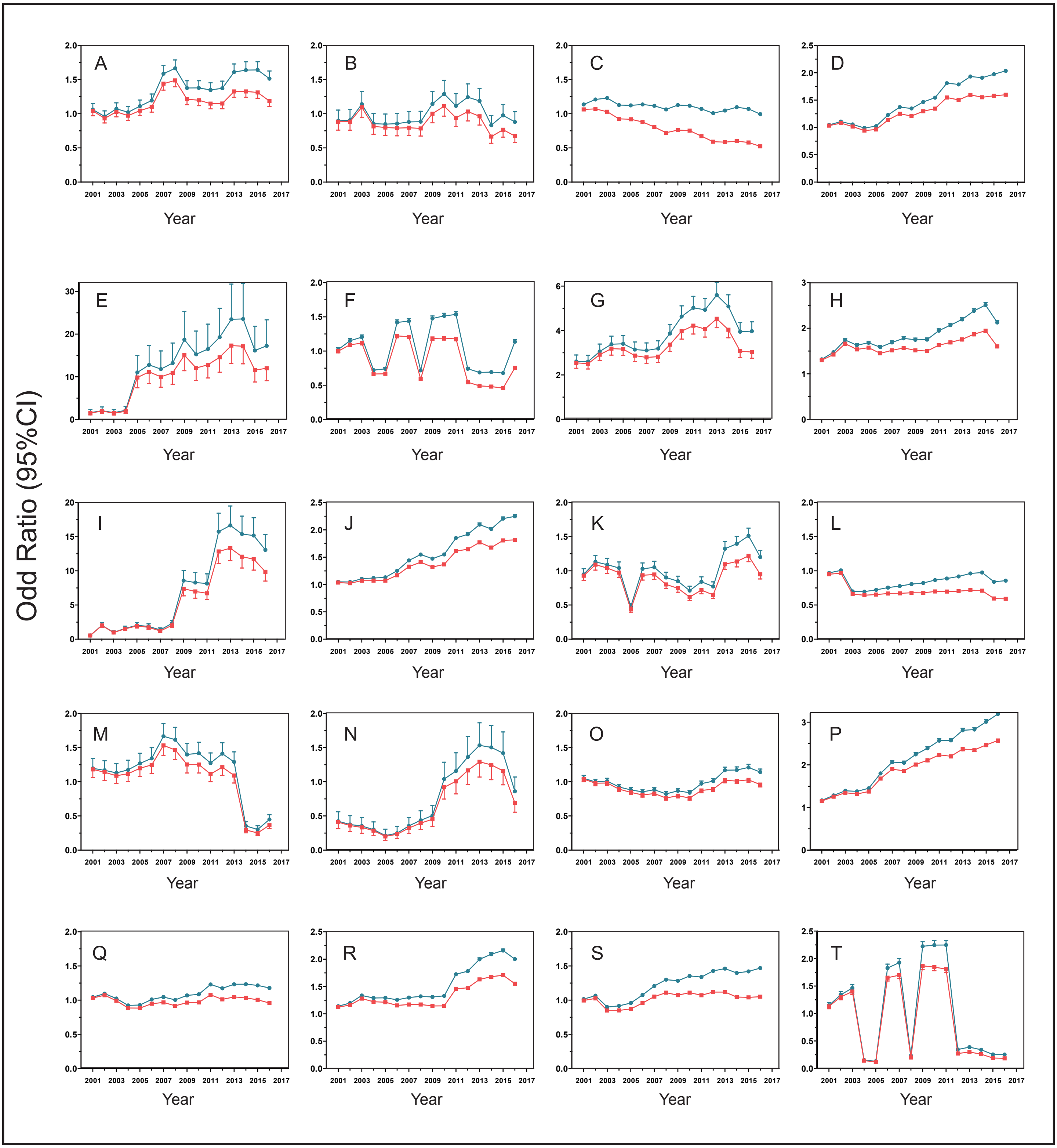

Appendix S15. Age-standardized odd ratio of rates compared to year 2000, by different subgroups

Note: ***A-E,*** by complication types, A: Neurological, B: Ophthalmic, C: Peripheral circulatory, D: Renal; ***E-L***, by regions, E: Africa, F: Asia, G: Caribbean, H: Europe, I: Middle east, J: North America, K: Oceania, L: South America; ***M-Q***, by DM types, M: Malnutrition-related DM, N: Other specified DM, O: T1DM, P: T2DM, Q: Unspecified DM; ***R-T***, by domestic income, R: High, S: Upper middle, T: Lower middle

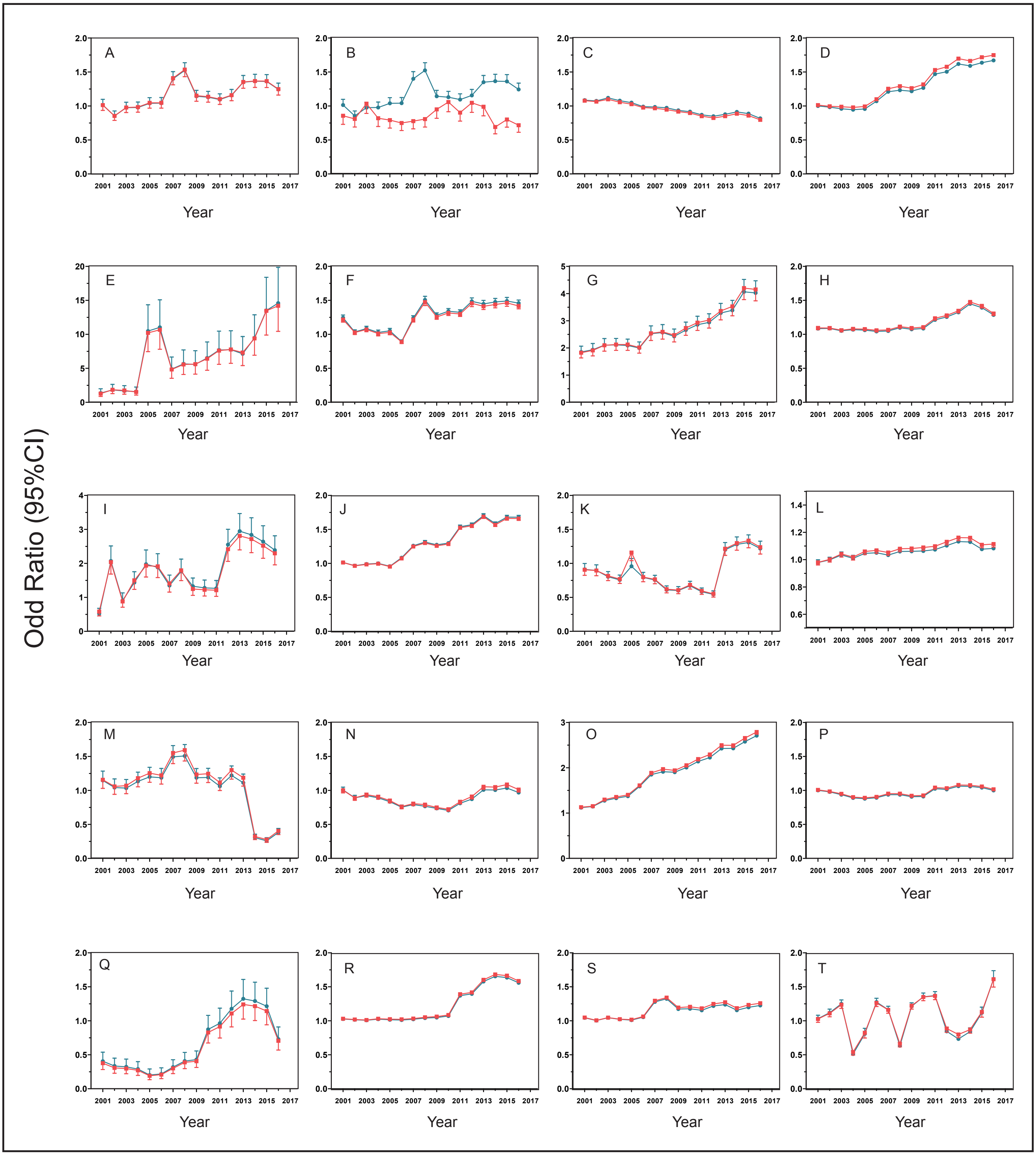

Appendix S16 . Age-standardized odd ratio of proportions compared to year 2000, by different subgroups

Note: ***A-E,*** by complication types, A: Neurological, B: Ophthalmic, C: Peripheral circulatory, D: Renal; ***E-L***, by regions, E: Africa, F: Asia, G: Caribbean, H: Europe, I: Middle east, J: North America, K: Oceania, L: South America; ***M-Q***, by DM types, M: Malnutrition-related DM, N: Other specified DM, O: T1DM, P: T2DM, Q: Unspecified DM; ***R-T***, by domestic income, R: High, S: Upper middle, T: Lower middle

Apendix S17. Country codes and names

Code	Country	Code	Country	Code	Country
**1300**	Mauritius	**2445**	Turks and Caicos Islands	**4240**	Portugal
**1303**	Mayotte	**2450**	United States of America	**4260**	Republic of Moldova
**1310**	Morocco	**2455**	Virgin Islands (USA)	**4270**	Romania
**1360**	Reunion	**2460**	Uruguay	**4273**	Serbia
**1365**	Rodrigues	**2470**	Venezuela	**4274**	Slovakia
**1520**	Tunisia	**3030**	Brunei Darussalam	**4280**	Spain
**2005**	Anguilla	**3080**	Cyprus	**4290**	Sweden
**2010**	Antigua and Barbuda	**3090**	Hong Kong SAR	**4300**	Switzerland
**2020**	Argentina	**3150**	Israel	**4308**	United Kingdom
**2025**	Aruba	**3160**	Japan	**4310**	United Kingdom, England and Wales
**2030**	Bahamas	**3170**	Jordan	**4320**	United Kingdom, Northern Ireland
**2040**	Barbados	**3190**	Kuwait	**4330**	United Kingdom, Scotland
**2045**	Belize	**3255**	Maldives	**4350**	Serbia and Montenegro, Former
**2050**	Bermuda	**3260**	Mongolia	**5020**	Australia
**2060**	Bolivia	**3285**	Oman	**5070**	Fiji
**2070**	Brazil	**3300**	Philippines	**5150**	New Zealand
**2090**	Canada	**3325**	Republic of Korea		
**2110**	Cayman Islands	**3340**	Saudi Arabia		
**2120**	Chile	**3350**	Singapore		
**2130**	Colombia	**3365**	Sri Lanka		
**2140**	Costa Rica	**3380**	Thailand		
**2150**	Cuba	**3400**	Turkey		
**2160**	Dominica	**4007**	Armenia		
**2170**	Dominican Republic	**4010**	Austria		
**2180**	Ecuador	**4020**	Belgium		
**2190**	El Salvador	**4030**	Bulgaria		
**2210**	French Guiana	**4038**	Croatia		
**2230**	Grenada	**4045**	Czech Republic		
**2240**	Guadeloupe	**4050**	Denmark		
**2250**	Guatemala	**4055**	Estonia		
**2260**	Guyana	**4080**	France		
**2270**	Haiti	**4084**	Georgia		
**2280**	Honduras	**4085**	Germany		
**2290**	Jamaica	**4140**	Greece		
**2300**	Martinique	**4150**	Hungary		
**2310**	Mexico	**4160**	Iceland		
**2340**	Nicaragua	**4170**	Ireland		
**2350**	Panama	**4180**	Italy		
**2360**	Paraguay	**4184**	Kyrgyzstan		
**2380**	Puerto Rico	**4186**	Latvia		
**2385**	Saint Kitts and Nevis	**4188**	Lithuania		
**2400**	Saint Lucia	**4190**	Luxembourg		
**2410**	Saint Pierre and Miquelon	**4200**	Malta		
**2420**	Saint Vincent and Grenadines	**4210**	Netherlands		
**2430**	Suriname	**4220**	Norway		
**2440**	Trinidad and Tobago	**4230**	Poland		

Country names codes was derived from the WHO mortality database
https://www.who.int/healthinfo/statistics/mortality_rawdata/en/
